# A Comparative Assessment of Epidemiologically Different Cutaneous Leishmaniasis Outbreaks in Madrid, Spain and Tolima, Colombia: An Estimation of the Reproduction Number via a Mathematical Model

**DOI:** 10.3390/tropicalmed3020043

**Published:** 2018-04-19

**Authors:** Anuj Mubayi, Marlio Paredes, Juan Ospina

**Affiliations:** 1 Simon A. Levin Mathematical Computational and Modeling Sciences Center, School of Human Evolution and Social Change, Arizona State University, Tempe, AZ 85281, USA; 2 Departamento de Matemáticas, Universidad Militar Nueva Granada, Sede Campus Nueva Granada, kilómetro 2 vía Cajicá—Zipaquirá, Cajicá, Colombia; marlio.paredes@unimilitar.edu.co; 3 Instituto de Ciencia Tecnología e Innovación, Universidad Francisco Gavidia, Calle El Progreso No. 2748, Edificio de Rectoría, San Salvador, El Salvador; 4 Department of Mathematics-Physics and Institute of Interdisciplinary Research, University of Puerto Rico at Cayey, 205 Antonio R. Barceló, Cayey, PR 00736, USA; 5 Eafit University, Carrera 49 N 7 Sur-50, Medellín, Colombia; jospina65@gmail.com

**Keywords:** cutaneous leishmaniasis, visceral leishmaniasis, mathematical modeling, SIR model, transmission dynamics of CL, health disparities, movement of individuals, multiple hosts

## Abstract

Leishmaniasis is a neglected tropical disease caused by the *Leishmania* parasite and transmitted by the Phlebotominae subfamily of sandflies, which infects humans and other mammals. Clinical manifestations of the disease include cutaneous leishmaniasis (CL), mucocutaneous leishmaniasis (MCL) and visceral leishmaniasis (VL) with a majority (more than three-quarters) of worldwide cases being CL. There are a number of risk factors for CL, such as the presence of multiple reservoirs, the movement of individuals, inequality, and social determinants of health. However, studies related to the role of these factors in the dynamics of CL have been limited. In this work, we (i) develop and analyze a vector-borne epidemic model to study the dynamics of CL in two ecologically distinct CL-affected regions—Madrid, Spain and Tolima, Colombia; (ii) derived three different methods for the estimation of model parameters by reducing the dimension of the systems; (iii) estimated reproduction numbers for the 2010 outbreak in Madrid and the 2016 outbreak in Tolima; and (iv) compared the transmission potential of the two economically-different regions and provided different epidemiological metrics that can be derived (and used for evaluating an outbreak), once *R*_0_ is known and additional data are available. On average, Spain has reported only a few hundred CL cases annually, but in the course of the outbreak during 2009–2012, a much higher number of cases than expected were reported and that too in the single city of Madrid. Cases in humans were accompanied by sharp increase in infections among domestic dogs, the natural reservoir of CL. On the other hand, CL has reemerged in Colombia primarily during the last decade, because of the frequent movement of military personnel to domestic regions from forested areas, where they have increased exposure to vectors. In 2016, Tolima saw an unexpectedly high number of cases leading to two successive outbreaks. On comparing, we estimated reproduction number of the Madrid outbreak to be 3.1 (with range of 2.8–3.9), which was much higher than reproduction number estimates of the Tolima first outbreak 1.2 (with range of 1.1–1.3), and the estimate for the second outbreak in Tolima of 1.019 (with range of 1.018–1.021). This suggests that the epidemic outbreak in Madrid was much more severe than the Tolima outbreak, even though Madrid was economically better-off compared to Tolima. It indicates a potential relationship between urban development and increasing health disparities.

## 1. Introduction

Background: Leishmaniasis is a disease caused by an intracellular protozoan parasite (genus *Leishmania*), which is transmitted by the bite of a female phlebotomine sandfly. The clinical spectrum of leishmaniasis ranges from a self-resolving cutaneous ulcer to a lethal visceral illness. Cutaneous leishmaniasis (CL) is the most common form of leishmaniasis and causes skin lesions on the exposed parts of the body, leaving scars for life. About 95% of CL cases occur in the Americas, the Mediterranean, the Middle East and Central Asia [1]. More than two-thirds of new cases of CL occur in six countries: Afghanistan, Algeria, Brazil, Colombia, Iran and Syria. An estimated 0.7 million to 1.3 million new cases occur worldwide annually [1,2]. Anthroponotic CL (where humans are the major reservoir of the parasite) is predominantly urban and periurban and shows patterns of spatial clustering similar to those of anthroponotic visceral leishmaniasis (VL) in South-East Asia. The epidemiology of CL is complex, with intra- and inter-specific variation in transmission cycles, reservoir hosts, sandfly vectors, clinical manifestations and response to therapy, and there are multiple circulating *Leishmania* species in the same geographical area [1,2,3,4].

Leishmaniasis epidemiology in Spain: Human leishmaniasis in the Mediterranean basin, including Spain, is an endemic zoonotic disease. In Spain, the vector involved in the transmission of the leishmania parasite is a sandfly of the *Phlebotomus* genus (primarily *P. perniciosus*), which is active between May and October, and dogs are the main reservoir [5,6]. During 2000 to 2009, an average of 20 leishmaniasis cases was reported per year in the Madrid autonomous community (with an annual incidence rate of around 0.5 per 100,000 inhabitants) [5]. However, during the last quarter of 2010, a fivefold increase in the number of cases was detected, compared with the number seen in the previous years. Subsequent research confirmed that an outbreak of leishmaniasis started in July 2009 in the south-west area of the region of Madrid, mainly affecting four geographically close municipalities [7]. The surveillance system for canine leishmaniasis did not detect any increase in prevalence during the period. Improvements in sanitation and disinfection in affected areas were also carried out as control measures [5]. Xenodiagnosis studies found that hares may have played a role as active reservoirs for the leishmania parasite [8]. The discovery of the new reservoir initially posed a challenge for controlling the outbreak. Rabbits were also known to be sources of blood meal for the vector species before this outbreak. Although dogs are the main reservoir host, hares are suspected to be a potential culprit for the surprising increase of cases during this outbreak. This was the largest reported community outbreak of leishmaniasis in Europe, despite Spain being one of the most economically-developed nations in the world, with continued intervention measures to control the disease [5].

Leishmaniasis epidemiology in Colombia: Colombia is one of two countries in the America region with highest number of leishmaniasis cases [1,9]. In 2016, about 10,743 new cases of leishmaniasis were reported from the country [10]. About 99.3% of all cases that occur in Colombia are CL, and the rest are infected with other forms of leishmaniasis [11]. The disease prevails in much of the country, moving from sylvatic to domestic cycles; parasites that are in jungle scenarios reach urban areas due to human movement [1,12]. CL outbreaks caused by *L. braziliensis*, *L. panamensis* and *L. guyanensis* are associated with intra- and peri-domiciliary transmission, which have been reported since 1984 [1,13,14]. Ramirez et al. [1] using data collected from 1980 to 2001, confirmed the leishmania species that caused CL in Colombia, and found *L. panamensis* (61.3%, 201 of 327 isolates), *L. braziliensis* (27.1%, 88/327), *L. infantum chagasi* (4%, 12/327), *L. mexicana* (2.1%, 8/327), and *L. amazonensis* (2.8%, 9/327) to be the primary species. In Colombia, CL is the most common manifestation in army personnel, who are the most vulnerable population, due to the continuous deployment of troops to forested areas of high endemicity and high density of the insect vector [15]. 

Health disparities: A central aspect of disparities is to identify and study differences in health status between groups, which negatively impact less advantaged groups. These differences could be because of socioeconomic status, gender and ethnicity disparities, and accessibility to health care and interventions. For example, despite the United States’ economic dominance and status as one of the most developed countries, an estimated 12 million Americans living in poverty suffer from at least one neglected tropical disease (NTD) [16]. While there are immense challenges to systematically investigating the potential impact of health disparities on an outbreak, overall potential of transmission and size of an infection for distinct populations can be estimated via metrics such as reproduction number, inoculation rate, epidemic size, vectorial capacity, and so forth. In this study, we modeled leishmaniasis outbreaks in the regions of Madrid, Spain and in Tolima, Colombia, as two ‘distinct’ populations to study differences in transmission potential. Spain's healthcare system is regularly rated among the world’s best (with ~90% of patients accessing public healthcare and ~20% accessing some part of private healthcare) and spends about 10% of its gross domestic product (GDP) on healthcare. State healthcare guarantees universal coverage, although one may have to travel far to find, or wait a significant time to access, a public healthcare facility. On the other hand, Colombia ranks 22nd on the WHO’s list of the best healthcare systems, with private healthcare establishments accounting for around 57% of establishments (thus, relatively rapid access to healthcare). Total expenditures on health constitute around 7% of Colombia’s GDP; however, urban and rural areas have significant differences in access to health care (see Table in the Supplementary Material, Section S.1, for details on potential health disparity between Spain and Colombia). Here, we do not aim to identify (or study) specific factors for health disparity leading to a leishmaniasis epidemic. However, a general discussion of the potential impact of health disparity on the disease outbreak is provided. Significant social and environmental data are needed to truly capture the differences and study the role of health disparity in the transmission dynamics of CL.

Mathematical modeling study of leishmaniasis and reproduction number: Initial mathematical models of CL transmission dynamics were developed by Dye et al. [17,18], in which they analyzed simple discrete-time epidemic models to study the mechanism behind observed inter-epidemic periods and the intensity of infection in dog reservoirs. Other studies included models with heterogeneous biting among age-structured dog populations and used serological data for the dog population in Gozo, Malta to estimate the basic reproduction number [19], a quantity that measures the intensity of an outbreak. Formally, the basic reproduction number (*R*_0_) of an infection can be interpreted as the average number of new cases generated by a typical infectious individual over the course of its infectious period, in an otherwise uninfected population. It is a key parameter, the value of which characterizes the transmission potential of an epidemic and hence, is often used to inform the potential effectiveness of intervention strategies. There are various ways to estimate *R*_0_ [20,21]. Here, we derive three novel methods to estimate *R*_0_ for a CL outbreak via a mathematical model with region-dependent features. The methods are tested using data from two ecologically distinct regions—Madrid (a city in a ‘developed’ country, Spain) and Tolima (a city in a ‘developing’ country, Colombia)—as a case study. In the literature, models have especially been used to estimate *R*_0_ [18] (see Table 1 for review on *R*_0_ estimates); however, such studies have used data primarily from different unrelated studies to collect point estimates of model parameters instead of applying a rigorous parameter estimation procedure. In the present study, we developed mathematical procedures for the estimation of model parameters via fitting the model to temporal incidence data, using three different techniques.

Research focus of the study: This study attempts to understand three major CL outbreaks, the first one in Madrid, Spain from 2009 to 2012 and the second and third outbreaks in Tolima, Colombia—both occurred in 2016. The outbreak in Madrid was started mainly by dogs, which are reservoir hosts of the disease, and the outbreaks in Tolima initiated because of the movement of soldiers, particularly those coming from the jungle to urban areas after staying in forestlands for long periods of time. In this work, we study the dynamics of CL in Spain and Colombia using a simple vector-borne disease model, while incorporating local characteristics and data on the disease. We used the country-specific model to estimate the transmission potential of each of three outbreaks via three novel parameter estimation procedures. These two regions were selected because of their distinct characteristics related to the disease and to highlight the comparison of the transmission potential between the ecologically and economically different regions. The estimated model parameters were then used to estimate the local reproduction number for each outbreak and each region. The difference in the basic reproduction number between regions could shed light on potential differences in health inequality, population immunity and transmissibility of leishmaniasis. This information is also important for designing effective control policies.

## 2. Materials and Methods 

**Figure 1:**
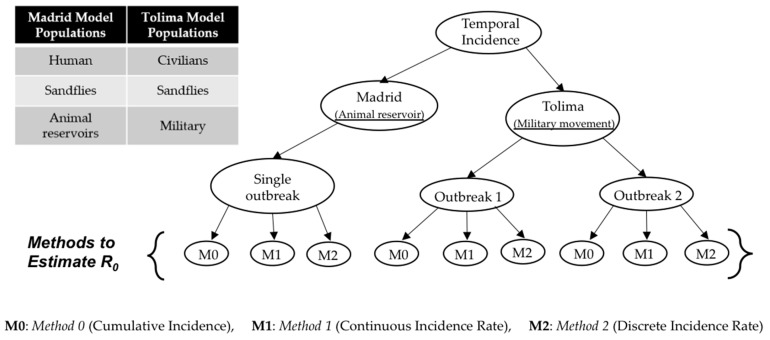
Types of models and estimation methods used in this article.

We used outbreak specific-models for the two regions—Madrid and Tolima—and estimated their parameters using three different estimation methods (methodologies shown in Figure 1; details of the methods are provided in later sections).

### 2.1. Data Sources

#### 2.1.1. Data Sources for Madrid, Spain

**Figure 2:**
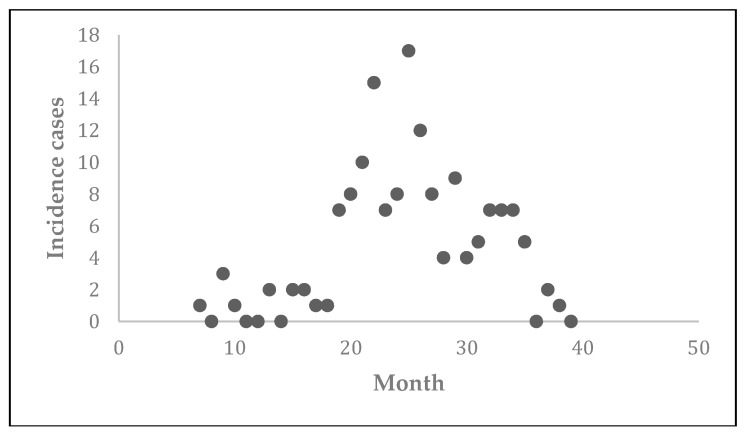
Reported number of new cases of cutaneous leishmaniasis (CL) from southwest communities of Madrid from July 2009 to March 2012.

The data corresponding to the 2009–2012 CL outbreak in communities of Madrid, Spain were collected (Figure 2) for the study by Mar Noguerol Álvarez et al. [30]. These reported data consisted of a monthly collection of new cases over the four-year period. The epidemic started in July 2009 (7th month of the year), ended in March 2012 and resulted in 156 total cases. The patients were primarily reported from four communities, namely, Fuenlabrada, Leganés, Getafe, and Humanes de Madrid. 

The mean incidence was found to be 14 cases per 100,000 inhabitants during this period. The incidence rate during the 2009 to 2012 epidemic was much higher than the corresponding rate during the 2000 to 2009 period from the same regions (between 1 and 6 new cases per year or incidence rate of less than 1 per 100,000 inhabitants). More than 60% of reported cases were male with a mean age of 46 years. *L. infantum* was identified as the causative pathogen species. It is important to note that around one quarter of cases had contact with dogs in one or more places in the domestic or peridomestic environment. The present study uses these 2009–2012 epidemic data from Madrid and estimates the basic reproduction number for the outbreak using a mathematical model.

#### 2.1.2. Data Sources for Tolima, Colombia

**Figure 3:**
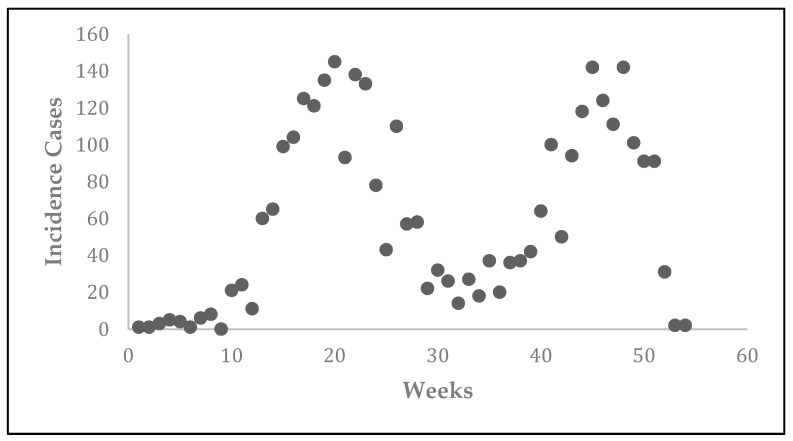
Reported number of new cases of CL from Tolima, Colombia during 2016.

The CL epidemic was observed in Tolima, Colombia in 2016 (Figure 3). The epidemic data representing new cases per week were obtained from the National Institute of Health’s Weekly Bulletin [10]. The outbreak started during the first week of January of 2016 (epidemiological week 1) and ended during the first week of January of 2017 (epidemiological week 53). There were two consecutive outbreaks resulting in 3223 total reported cases. The second outbreak might have been a result of heavy movement of troops back to the city from forest areas as a result of peace deal signed between government and Revolutionary Armed Forces of Colombia (FARC) rebels. The first outbreak occurred between week 1 and week 35 and the second outbreak occurred between week 36 and week 53. The highest number of reported cases (~149) occurred on 15 May (~week 20) and 15 November (~week 46). In this study, we use these two outbreaks in Tolima and estimate the corresponding basic reproduction numbers using a mathematical model.

### 2.2. Model Description

#### 2.2.1. Mathematical Model for CL Epidemic in Madrid, Spain

**Figure 4:**
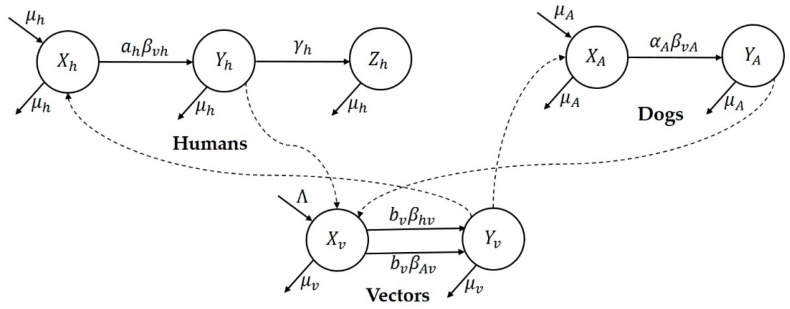
Flow chart representing the mathematical model for Madrid.

Since dogs are the major reservoir of the disease in Spain, we considered a transmission dynamics model consisting of human (represented by subscript ‘h’), dogs (represented by subscript ‘A’), and sandfly vector (represented by subscript ‘v’) populations [16]. The susceptible, infectious and recovered subcategories for the three populations are represented by X, Y, and Z, respectively. The flowchart of the epidemic model for the Madrid outbreak is illustrated in Figure 4. The model equations are given in Supplementary Material (Section S.2) and state variables and parameters are explained in Table 2.

Here, $N_{h},N_{v}$ and $N_{A}$ represent the total population of humans, vectors and dogs, respectively. We assume these populations to be constant by taking equal natural birth and death rates (where, $\mu_{h},\mu_{v}$ and $\mu_{A}$ represented both birth and death per capita rates for the three populations). The probability of a vector choosing a human to bite is $\frac{N_{h}}{N_{h}+N_{A}}$ and therefore, a human receives $a_{h}\frac{N_{v}}{N_{h}}\frac{N_{h}}{N_{h}+N_{A}}$ bites per unit time and a vector takes $a_{h}\frac{N_{h}}{N_{h}+N_{A}}$ human blood meals per unit time. Hence, the infection rates per susceptible human and susceptible vector are given by

$$\beta_{vh}a_{h}\frac{N_{v}}{N_{h}}\frac{N_{h}}{N_{h}+N_{A}}\frac{Y_{v}}{N_{v}}=\frac{\beta_{vh}a_{h}}{N_{h}+N_{A}}Y_{v}\;\mathrm{and}\;\beta_{hv}b_{v}\frac{N_{h}}{N_{h}+N_{A}}\frac{Y_{h}}{N_{h}}=\frac{\beta_{hv}b_{v}}{N_{h}+N_{A}}Y_{h},$$

respectively. Similarly, the probability that a vector bites a dog is $\frac{N_{A}}{N_{h}+N_{A}}$. Hence, a dog receives $\alpha_{a}\frac{N_{v}}{N_{A}}\frac{N_{A}}{N_{h}+N_{A}}$ bites per unit time and a vector takes $\alpha_{a}\frac{N_{A}}{N_{h}+N_{A}}$ dog blood meals per unit time. Hence, the infection rates per susceptible dog and susceptible vector are given by

$$\beta_{vA}\alpha_{A}\frac{N_{v}}{N_{A}}\frac{N_{A}}{N_{h}+N_{A}}\frac{Y_{v}}{N_{v}}=\frac{\beta_{vA}\alpha_{A}}{N_{h}+N_{A}}Y_{v}\;\mathrm{and}\;\beta_{Av}b_{v}\frac{N_{A}}{N_{h}+N_{A}}\frac{Y_{A}}{N_{A}}=\frac{\beta_{Av}b_{v}}{N_{h}+N_{A}}Y_{A},$$

respectively. The corresponding model’s equations are included in the Supplementary Material (Section S.2.1).

#### 2.2.2. Mathematical Model for CL Epidemic in Tolima, Colombia

**Figure 5:**
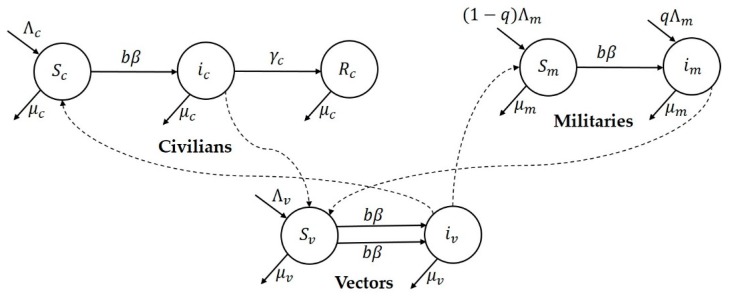
Flow chart representing the mathematical model for Tolima.

Since the movement of military personal from the forest to civilian regions resulted in an unprecedented higher number of cases in Colombia, we considered the transmission dynamical model consisting of civilian (represented by subscript ‘c’), military (represented by subscript ‘m’) and sandfly vector (represented by subscript ‘v’) populations interacting with each other [16]. The susceptible, infectious and recovered subcategories are represented by S, i, and R, respectively. The model framework that is used for the two Tolima, Colombia outbreaks is illustrated in the flow chart in Figure 5. The model state variables and parameters are explained in Table 3.

The civilian or military susceptible population moves to the class of infected population by the bite of an infected vector biting at the rate b. Similarly, the susceptible vector population moves to the infectious class by the bite of a female vector to an infected civilian or military. For each population, the total recruitment rate is $\Lambda_{c},\Lambda_{m}$ and $\Lambda_{v}$ and the per capita death rates are $\mu_{c},\mu_{m}$ and $\mu_{v}$. The recovery rate for the civilian population is $\gamma_{c}$. It is assumed that proportion q of the traveling military individuals move to the town are infected and proportion (1-q) are assumed to be susceptible. The corresponding model’s differential equations are included in the Supplementary Material (Section S.2.2).

We assumed two different new incoming rate parameters each for civilian ($\Lambda_{c}$) and military ($\Lambda_{m}$) populations. The incoming rate for military incorporate two constant rates: the net recruitment rate in military from civilian population and the net movement rate from other surrounding areas to the modeled region of Tolima. The recruitment into military occurs at national (country) level. In urban areas of Tolima, Colombia, military populations are stationed in battalion camps, which are typically on the outskirts of the urban areas. These military camps have all relevant facilities but only limited interactions with civilians occur. This is to ensure safety of military individuals (as they fight with local guerillas, who are often friendly with civilians) and to maintain secrecy of military operations.

## 3. Analysis

The well-known Ross-Macdonald model, developed in 1911, formally initiated the field of modeling of complex transmission cycles of vector borne diseases [31] and provided a theoretical support for understanding the dynamics of those infections. The key quantities such as the basic reproduction number, vectorial capacity and inoculation rates derived from the analysis of vector-borne models become the central to the quantification of transmission. Once the model parameters are estimated, these quantities can be easily computed. Here, we develop the enhanced expression and estimate of some of these quantities using region-specific characteristics and data. 

### 3.1. Reproduction Numbers

The *type-reproduction number* (*R_T_*) for a specific host type is interpreted as the average number of secondary cases of that type produced by the primary cases of the same host type during the entire course of infection. It takes into account not only the secondary cases directly transmitted from the specific host but also the cases indirectly transmitted by way of other types, who were infected from the primary cases of the specific host with no intermediate cases of the target host. It is a useful measure when a particular single host type is targeted in the disease control effort in a community with various types of host [32]. R_T_ can be seen as an extension of *R*_0_ in a sense that the threshold condition of the total population growth can be formulated by the reproduction process of the target type only. 

**Figure 6:**
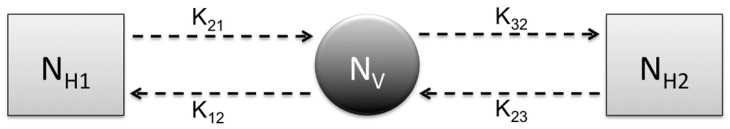
Flow chart of general vector borne model with two types of hosts H1 and H2 as well as one vector population.

The next generation matrix NGM using Figure 6 can be computed as $K=\left(\begin{array}{ccc} 0 & K_{12} & 0\\ K_{21} & 0 & K_{23}\\ 0 & K_{32} & 0 \end{array}\right)$, and hence, the basic reproduction number is $R_{0}=\rho \left(K\right)=\sqrt{K_{12}K_{21}+K_{32}K_{23}}=\sqrt{R_{0}^{h1}R_{0}^{v1}+R_{0}^{h2}R_{0}^{v2}}$, where $K_{ij}$ represents average number of new infections among the susceptible of type *i*, generated by an infected of type *j*. Note, for vector borne diseases, infected host of type 1 cannot directly infect susceptible host of type 3 and vice versa, and type 2 represents the vector. The type reproduction number for Type One is

$$R_{T1}=K_{21}K_{12}+K_{21}(K_{32}K_{23})K_{12}+K_{21}(K_{32}K_{23}\cdot K_{32}K_{23})K_{12}+\cdots =\frac{K_{12}K_{21}}{1-K_{32}K_{23}}$$

If $K_{32}K_{23}>1$ then the series fails to converge and Type Three hosts, $N_{H2}$, is a reservoir of infection. The type reproduction numbers of Type Two and Type Three, respectively, are $R_{T2}=K_{21}K_{12}+K_{32}K_{23}$ and $R_{T1}=\frac{K_{23}K_{32}}{1-K_{12}K_{21}}$.

The next-generation matrix (NGM), introduced by Diekmann et al. [33], provides a procedure to derive the *basic reproduction number*, *R*_0_. This matrix (often denoted by *K* = [*k_ij_*]) gives the average number of new infections among the susceptible individuals of type *i*, generated by an infected individual of type *j* and *R*_0_ is identified as its dominant eigenvalue (that is, *R*_0_ = *ρ*(*K*)). In some special models, *K = F × V*^−1^, where *F* is the new generation matrix and *V* represents the transition matrix [34]. However, this is not true for vector-borne models with multiple hosts, as is the case in this study. Note, $R_{0}<1\;iff\;R_{Ti}$ for all host type *i* and if $R_{Ti}>1$ then host type i is a reservoir of infection. 

For the *K* = [*k_ij_*], one identifies the set of targeted entries *S*, that is, the set of entries in *K* that are subject to change in control. The target matrix K_S_ is identified as [*K_S_*]*_ij_* = *k_ij_* if (*i*,*j*) ∈ *S*, and zero otherwise. The *target reproduction number, R_S_* is defined as *R_S_* = *ρ*(*K_S_*·(*I* − *K* + *K_S_*)^−1^) provided that *ρ*(*K* − *K_S_*) < 1, where I is the identity matrix [35]. The last condition can be referred to as the condition for controllability, since if the spectral radius is greater than 1 then the disease cannot be eliminated by targeting only *S* (in such case, *R_S_* is not defined [36]). The *controlled NGM*, *K_c_*, is formulated by replacing the entry *k_ij_* in *K* by *k_ij_*/*R_S_* whenever (*i*,*j*) ∈ *S*.

Typically, in the case of the simple one-host vector-borne epidemic model, the computed basic reproduction number is given by $R_{0}^{2}=R_{0}^{h}R_{0}^{v}$, where $R_{0}^{h}$ represents average number of human cases generated by one vector and $R_{0}^{v}$ is the average number of vector cases generated by one host. Therefore, the basic reproduction number *R*_0_ gives the average number of secondary infectious hosts (or vectors) produced by one primary infectious hosts (or vector) introduced in completely susceptible populations of hosts and vectors. The *effective reproduction number* $R_{eff}\left(t\right)$ can be defined as product of partial effective reproduction numbers $R_{0}^{h}\cdot \left(S_{h}\left(t\right)/N_{h}\left(t\right)\right)$ and $R_{0}^{v}\cdot \left(S_{v}\left(t\right)/N_{v}\left(t\right)\right)$ as

(1) $$R_{eff}\left(t\right)=\left(R_{0}^{h}\cdot \frac{S_{h}\left(t\right)}{N_{h}\left(t\right)}\right)\cdot \left(R_{0}^{v}\cdot \frac{S_{v}\left(t\right)}{N_{v}\left(t\right)}\right)$$

where $S_{h}\left(t\right),N_{h}\left(t\right),S_{v}\left(t\right),\;and\;N_{v}\left(t\right)$ represents number of susceptible hosts, total size of host population, density of susceptible vectors, total density of vector population, respectively. At the time of the beginning of epidemics, $R_{eff}=R_{0}$ because all hosts and vectors in their respective populations are susceptible. Moreover, for large time, the epidemics reaches a steady state, which occurs due to $R_{eff}=1.$ Also, *R_eff_* formula could be used to numerically see the difference in Madrid and Tolima outbreaks over time. 

### 3.2. Mathematical Computations

#### 3.2.1. Mathematical Computations for Spain Model

The basic reproductive number for the model (S1)–(S7) has the form

(2) $$R_{0}^{2}=\frac{\beta_{hv}b_{v}N_{h}}{\left(N_{h}+N_{A}\right)\left(\mu_{h}+\gamma_{h}\right)}\frac{\beta_{vh}a_{h}\Lambda /\mu_{v}}{\left(N_{h}+N_{A}\right)\mu_{v}}+\frac{\beta_{Av}b_{v}N_{A}}{\left(N_{h}+N_{A}\right)\mu_{A}}\frac{\beta_{vA}\alpha_{A}\Lambda /\mu_{v}}{\left(N_{h}+N_{A}\right)\mu_{v}}.$$

We transform the complex model (S1)–(S7) and we obtain the following effective simple epidemic model

(3) $$\frac{dX_{h}}{dt}=-\beta_{eff}X_{h}Y_{h}$$

(4) $$\frac{dY_{h}}{dt}=\beta_{eff}X_{h}Y_{h}-\gamma_{eff,h}Y_{h}$$

(5) $$\frac{dZ_{h}}{dt}=\gamma_{eff,h}Y_{h}$$

where $\gamma_{eff,h}=\gamma_{h}+\mu_{h}$ and

(6) $$\beta_{eff}=\frac{a_{h}\beta_{vh}b_{v}\beta_{hv}N_{v}\mu_{A}}{\mu_{A}\mu_{v}N_{h}^{2}+2\mu_{A}\mu_{v}N_{h}N_{A}+\mu_{A}\mu_{v}N_{A}^{2}-b_{v}\beta_{Av}N_{v}\alpha_{a}\beta_{vA}N_{A}}.$$

The basic reproduction number for this model is

(7) $$R_{0}^{2}=\frac{a_{h}\beta_{vh}N_{h}\beta_{hv}b_{v}N_{v}}{\mu_{v}{\left(N_{h}+N_{A}\right)}^{2}\left(\gamma_{h}+\mu_{h}\right)}+\frac{b_{v}N_{v}N_{A}\beta_{Av}\alpha_{A}\beta_{vA}}{\mu_{A}\mu_{v}{\left(N_{h}+N_{A}\right)}^{2}}$$

#### 3.2.2. Mathematical Computations for Colombia Model

The basic reproduction number for the model (S25)–(S31) has the form

(8) $$R_{0}^{2}=\frac{b^{2}\beta^{2}\Lambda_{v}\Lambda_{m}}{N_{v}\mu_{v}^{2}\mu_{m}\left(N_{c}+N_{m}\right)}+\frac{b^{2}\beta^{2}\Lambda_{v}\Lambda_{c}}{N_{v}\mu_{v}^{2}\mu_{c}\left(N_{c}+N_{m}\right)\left(\mu_{c}+\gamma_{c}\right)}.$$

We transform the complex model (S25)–(S31) into an effective simple SIR model and obtain

(9) $$\frac{dS_{c}}{dt}=-\beta_{eff}S_{c}i_{c}$$

(10) $$\frac{di_{c}}{dt}=\beta_{eff}S_{c}i_{c}-\gamma_{c}i_{c}$$

(11) $$\frac{dR_{c}}{dt}=\gamma_{c}R_{c}$$

and for the basic reproduction number we obtain

(12) $$R_{0}^{2}=\frac{\beta^{2}b^{2}N_{c}}{\mu_{v}\left(\mu_{c}+\gamma_{c}\right)\left(N_{c}+N_{m}\right)}+\frac{\beta^{2}b^{2}N_{m}}{\mu_{v}\left(\mu_{m}+\gamma_{m}\right)\left(N_{c}+N_{m}\right)}$$

Note, Equations (3)–(5) and (9)–(11) are similar.

### 3.3. Parameter Estimation Procedure

Three different methods were used to estimate model parameters and hence *R*_0_. Supplementary Material (Section S.3) contains the detailed explanation of the parameter estimation procedures and the corresponding codes.

#### 3.3.1. Method 0 (Cumulative Incidence Technique) 

Dividing Equation (3) by (5) and then integrating we can compute expression of $X_{h}\left(t\right)$ in terms of $Z_{h}\left(t\right)$. Since $Y_{h}\left(t\right)=1-X_{h}\left(t\right)-Z_{h}\left(t\right)$ and $X_{h}\left(t\right)$ function of $Z_{h}\left(t\right)$, Equation (5) can be written only in terms of single variable $Z_{h}\left(t\right)$. Expanding right hand side of this new equation using its Taylor series and approximating only up to quadratic terms. Finally, integrating it to obtain the solution of $Z_{h}\left(t\right)$.

That is, the SIR model (3)–(5) can be solved approximately (see detailed computations in Section 2.3.2 of [37]) as

(13) $$Z_{h}\left(t\right)=\frac{\rho^{2}\left(\frac{s}{\rho }-1-\alpha \tanh\left(-0.5\alpha \gamma t+\phi \right)\right)}{s}$$

where $s=N_{h}$, $\beta =\beta_{eff}$, $\gamma =\gamma_{eff,h}$,

(14) $$\alpha =\sqrt{{\left(\frac{s}{\rho }-1\right)}^{2}+\frac{2s}{\rho^{2}}}.$$

(15) $$\phi =\frac{1}{2}\ln\left(\frac{\alpha \rho +s-\rho }{\alpha \rho -s+\rho }\right).$$

(16) $$\rho =\frac{\gamma }{\beta }$$

Furthermore, $X_{h}\left(t\right)=\frac{X_{h}\left(0\right)e^{-Z_{h}\left(t\right)}}{\rho }$ and $Y_{h}\left(t\right)=1-X_{h}\left(t\right)-Z_{h}\left(t\right)$. Hence, cumulative incidence by time *t* is $\int_{0}^{t}\beta_{eff}X_{h}\left(l\right)Y_{h}\left(l\right)dl$ or simply $Z_{h}\left(t\right)$. Similarly, the cumulative incidence formula corresponding to the Equations (9)–(11)can be derived.

#### 3.3.2. Method 1 (Incidence Technique)

Taking the temporal derivative of (13) we obtain the theoretical incidence curve given by

(17) $$\frac{dZ_{h}}{dt}=\frac{1}{2}\frac{\rho^{2}\alpha^{2}\gamma }{s\cosh{\left(-\frac{\alpha \gamma }{2}t+\phi \right)}^{2}}$$

#### 3.3.3. Method 2 (Incidence Technique) 

Another method to obtain the theoretical incidence is to use (13), discretize time and take the difference between the two successive times, namely

(18) $$\mathrm{Inc}\left(t\right)=\frac{\rho^{2}\left(\frac{s}{\rho }-1-\alpha \tanh\left(-\frac{\alpha \gamma }{2}t+\phi \right)\right)}{s}-\frac{\rho^{2}\left(\frac{s}{\rho }-1-\alpha \tanh\left(-\frac{\alpha \gamma }{2}\left(t-1\right)+\phi \right)\right)}{s}.$$

This equation can be simplified as

(19) $$\mathrm{Inc}\left(t\right)=\frac{\rho^{2}\alpha \left(\tanh\left(-\frac{\alpha \gamma }{2}t+\phi \right)-\tanh\left(-\frac{\alpha \gamma }{2}t+\frac{\alpha \gamma }{2}+\phi \right)\right)}{s}$$

### 3.4. Epidemiological Evaluation Metrics Using Components of R_0_

Once *R*_0_ is estimated, we can derive other epidemiological metrics for evaluation of an outbreak. However, additional data may be also needed to compute some of these metrics. We do not use these other metrics for comparison of the outbreaks in the two regions considered in this study. 

*Vectorial Capacity* (VC) describes the potential for a vector population to transmit a parasite and can be interpreted as the total number of potentially infectious bites that would eventually arise from all the vectors biting a single perfectly infectious (i.e., all vector bites result in infection) human on a single day. For the Madrid model and under the assumption of presence of only human host,

$$VC=\frac{mb_{v}^{2}\beta_{hv}\beta_{vh}p^{n}}{-\ln\left(p\right)}.$$

where m is the number of vectors per hosts (i.e., *N_v_*/*N_h_*), n is the extrinsic incubation period and $p=e^{-\mu_{v}}$ is the probability of a mosquito surviving a day. In fact, $VC=\frac{R_{0}\gamma_{h}}{\beta_{vh}}$ can be written as a function of *R*_0_ [38].

The *Entomological Inoculation Rate* (EIR) is in general defined as the number of infectious bites per person per unit time and can be computed by multiplying the human biting rate with the fraction of infectious vector [39]. In other words, $EIR=\left(N_{v}b_{v}\right)\left(\frac{Y_{v}}{N_{v}}\right)=b_{v}Y_{v}$ for the Madrid model and under the assumption of presence of only human hosts. 

Often, defining the risk associated with a host or with a region is needed. There are two types of risk indexes commonly used for the vector borne diseases—the *Transmission Risk Index* and the *Vulnerability Risk Index*. The transmission risk index for host *i*, *TR_i_*, is defined as the probability *P_i_* that a host *i* gets infected, multiplied by the secondary cases it generate. Thus, it is given by $TR_{i}=P_{i}R_{0}^{hi}$, where $P_{i}=\frac{N_{i}}{\sum N_{k}}$ and summation is over only host populations. This index indicates the risk of the host *i* becoming the main transmitter of the infection at the beginning of an epidemic. On the other hand, we define the Vulnerability Risk index for host *j*, *VR_j_*, as the secondary infections in host *j* when another host of type *i* (≠*j*) becomes infected first at the beginning of an epidemic. Thus, $VR_{j}=R_{0}^{hj}\left(\sum_{i\neq j}P_{i}R_{0}^{vi}\right)$.

## 4. Results

Note, the next generation matrix NGM for the model in Figure 6 is $K=\left(\begin{array}{ccc} 0 & K_{12} & 0\\ K_{21} & 0 & K_{23}\\ 0 & K_{32} & 0 \end{array}\right)$. Hence, the NGM for the Spain model is

(20) $$\left(\begin{array}{ccc} humans\to humans & Humans\to vectors & humans\to dogs\\ vectors\to humans & vectors\to vectors & vectors\to dogs\\ dogs\to humans & dogs\to vectors & dogs\to dogs \end{array}\right)=\left(\begin{array}{ccc} 0 & K_{hv} & 0\\ K_{vh} & 0 & K_{vd}\\ 0 & K_{dv} & 0 \end{array}\right)$$

and for the Colombia model is

$$\left(\begin{array}{ccc} civilian\to civilian & civilian\to vectors & civilian\to military\\ vectors\to civilian & vectors\to vectors & vectors\to military\\ military\to civilian & military\to vectors & military\to military \end{array}\right)=\left(\begin{array}{ccc} 0 & K_{cv} & 0\\ K_{vc} & 0 & K_{vm}\\ 0 & K_{mv} & 0 \end{array}\right)$$

The components of the K matrix for our two models are given by:

Spain
$K_{12}=\frac{\beta_{vh}a_{h}N_{h}}{\left(N_{h}+N_{A}\right)\mu_{v}},K_{21}=\frac{\beta_{hv}b_{v}\left(\frac{\Lambda }{\mu_{v}}\right)}{\left(N_{h}+N_{A}\right)\left(\mu_{h}+\gamma_{h}\right)},\;K_{32}=\frac{\beta_{vA}\alpha_{A}N_{A}}{\left(N_{h}+N_{A}\right)\mu_{v}},\;K_{23}=\frac{\beta_{Av}b_{v}\left(\frac{\Lambda }{\mu_{v}}\right)}{\left(N_{h}+N_{A}\right)\mu_{A}}.$
Columbia
$K_{12}=\frac{b\beta (\Lambda_{c}/\mu_{c})}{(\Lambda_{v}/\mu_{v})\mu_{v}},K_{21}=\frac{b\beta (\Lambda_{v}/\mu_{v})}{\left(\left(\Lambda_{c}/\mu_{c})+(\Lambda_{m}/\mu_{m}\right)\right)\left(\mu_{c}+\gamma_{c}\right)},K_{32}=\frac{b\beta (\Lambda_{m}/\mu_{m})}{(\Lambda_{v}/\mu_{v})\mu_{v}},\;K_{21}=\frac{b\beta (\Lambda_{v}/\mu_{v})}{\left(\left(\Lambda_{c}/\mu_{c})+(\Lambda_{m}/\mu_{m}\right)\right)\mu_{m}}$


### 4.1. Estimates of $R_{0}$ for Madrid-Spain

**Figure 7:**
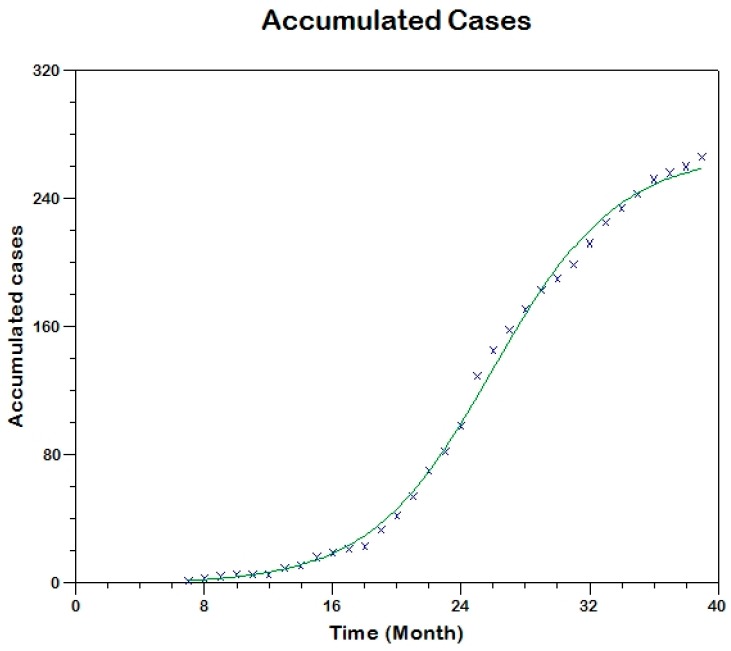
Model fit to the cumulative reported incidence of Madrid-Spain outbreak using Method 0.

**Figure 8:**
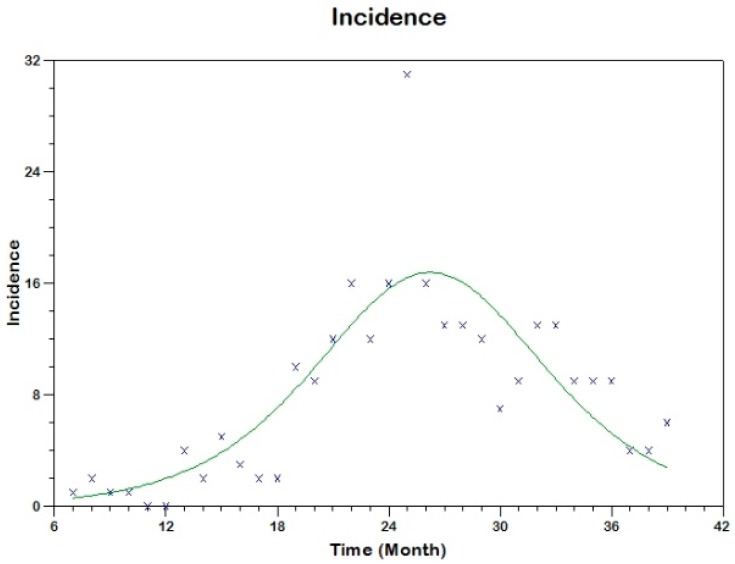
Model fit to the reported incidence of Madrid-Spain outbreak using Method 1.

We use equation (13) to fit the observed curve of accumulated cases to estimate the model parameters (that is, using Method 0; Figure 7) hence, obtaining

$$s_{h}\left(0\right)=711,\;\beta =0.00048,\;\gamma_{h}=0.0859,\;R_{0}^{2}=3.9$$

and we use equation (17) to fit the observed curve of incidence obtaining the following estimated values of the parameters (see Figure 8).

$$s_{h}\left(0\right)=629,\;\beta =0.00057,\;\gamma_{h}=0.1169,\;R_{0}^{2}=3.1$$

**Figure 9:**
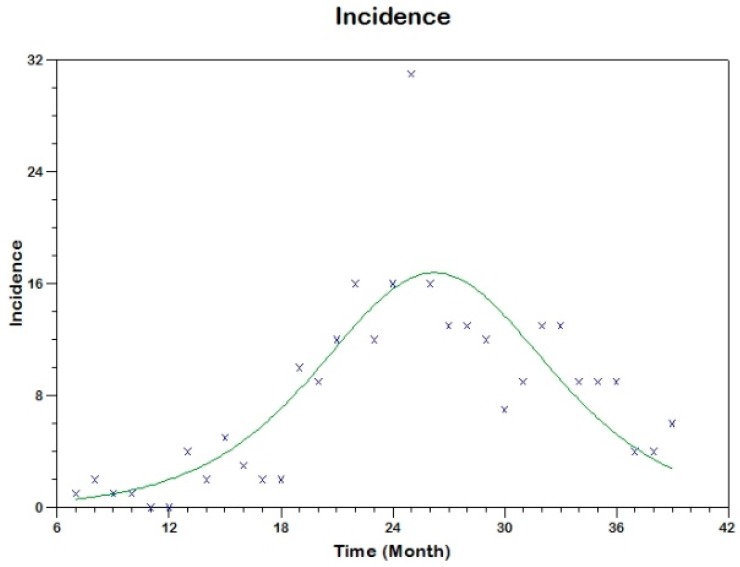
Model fit to the reported incidence of Madrid-Spain outbreak using Method 2.

We use equation (19) to fit the observed curve of incidence obtaining the following estimated values of the parameters (see Figure 9)

$$s_{h}\left(0\right)=606,\;\beta =0.00061,\;\gamma_{h}=0.1312,\;R_{0}^{2}=2.8$$

Table 4 summarizes the estimates obtained in this case.

The details of the estimation procedure and confidence interval are given in the Supplementary Material, Section S.3.1. The results suggest that around 626 individuals (with range of 606-711) were at-risk of CL in Madrid before 2009-2012 outbreak (see Table 4). The estimated reproduction number of the Madrid CL outbreak was found to be 3.1 with range from 2.8 to 3.9 (see Table 4).

### 4.2. Estimates of $R_{0}$ for Tolima-Colombia

#### 4.2.1. Tolima First Outbreak

**Figure 10:**
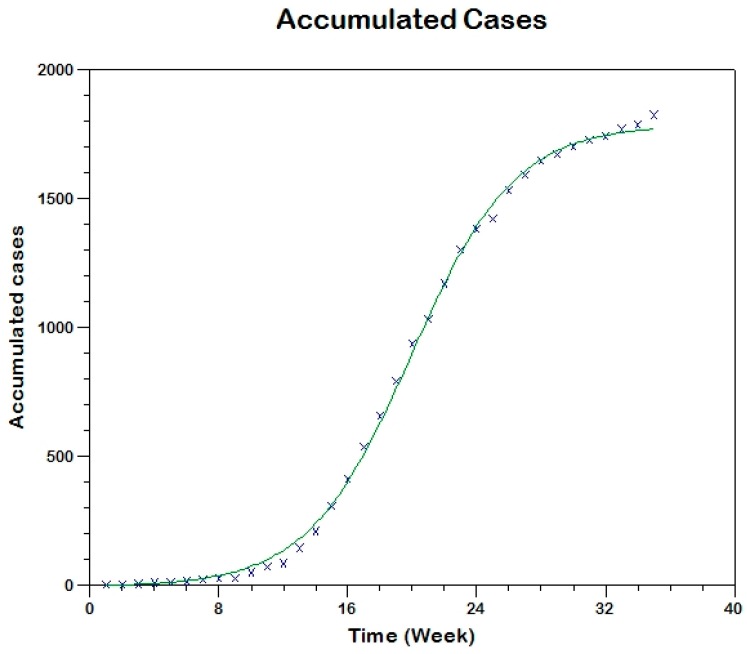
Model fit to the cumulative reported incidence of the first outbreak in Tolima-Colombia using Method 0.

**Figure 11:**
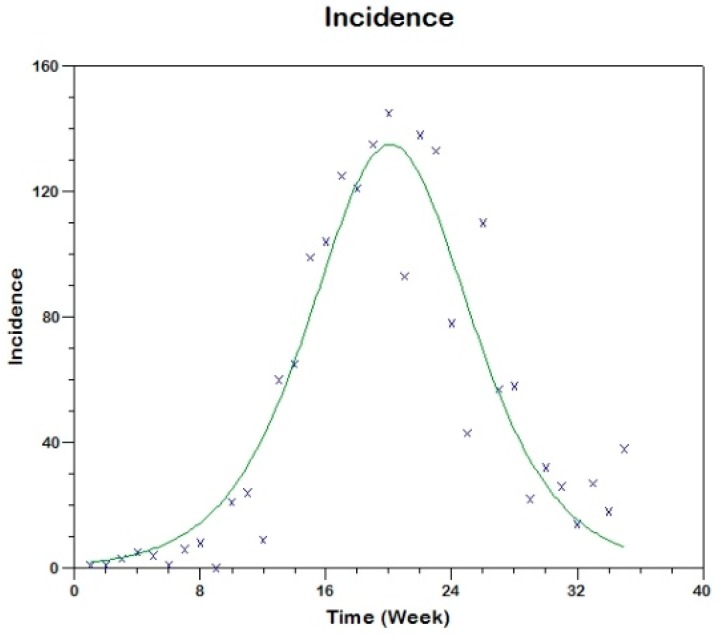
Model fit to the reported incidence rate of the first outbreak in Tolima-Colombia using Method 1.

**Figure 12:**
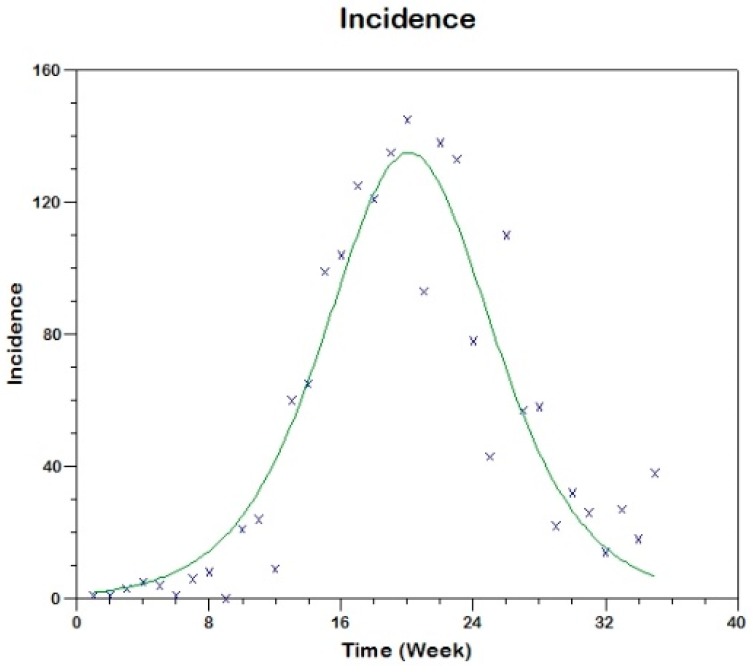
Model fit to the reported incidence of the first outbreak in Tolima-Colombia using Method 2.

In this case, we use similar methods to estimate the parameters of the respective model (see Supplementary Material Section S.3.2) obtaining the following results for the two outbreaks. The parameter estimation is carried out using three methods (the fitting are shown in Figure 10, Figure 11 and Figure 12).

The details of the estimation procedure and confidence interval are given in the Supplementary Material, Section S.3.2. The results suggest that around 6656 individuals (with range of 5288–7301) were at-risk of CL in Tolima-Colombia before 2016 outbreak (see Table 5). The estimated reproduction number of the Tolima CL outbreak was found to be 1.2 (see Table 5).

#### 4.2.2. Tolima Second Outbreak

**Figure 13:**
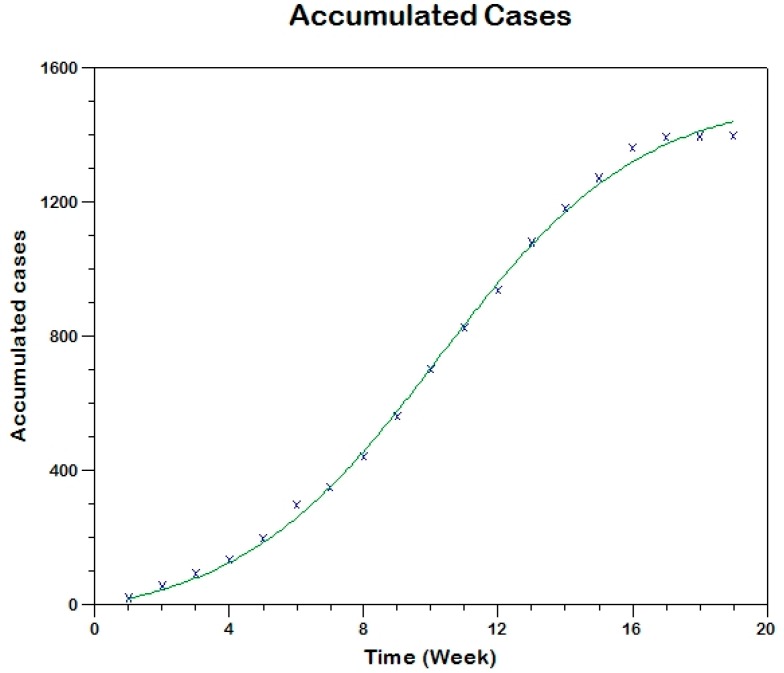
Model fit to the cumulative reported incidence of the second outbreak in Tolima-Colombia using Method 0.

**Figure 14:**
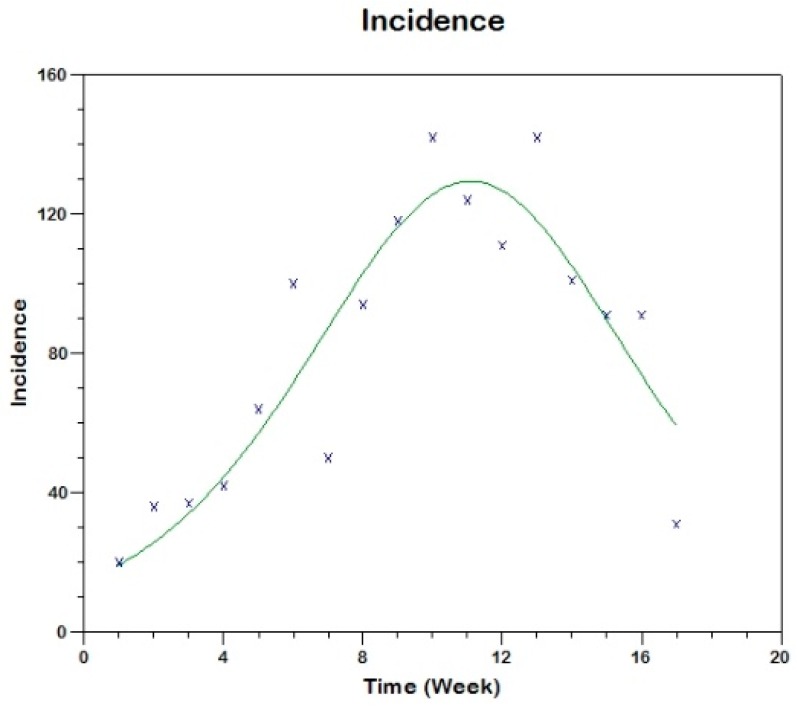
Model fit to the cumulative reported incidence of the second outbreak in Tolima-Colombia using Method 1.

**Figure 15:**
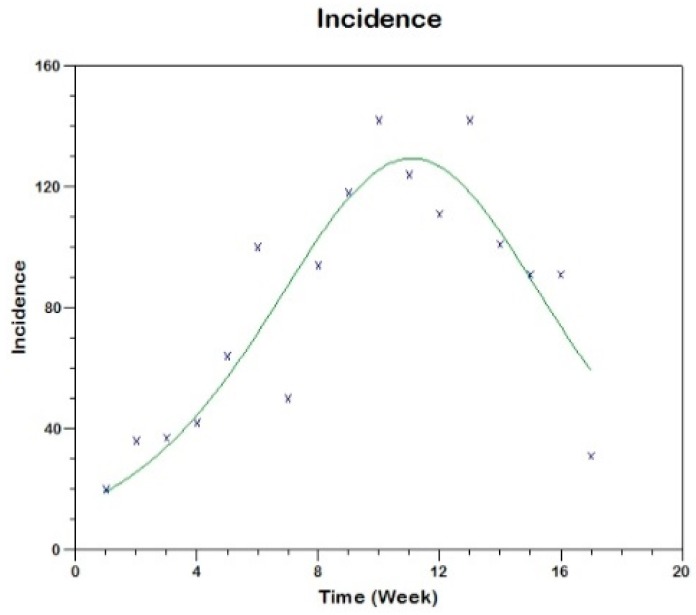
Model fit to the cumulative reported incidence of the second outbreak in Tolima-Colombia using Method 2.

In this case also the parameter estimation is carried out using the same three methods (the fitting are shown in Figure 13, Figure 14 and Figure 15).

The details of the estimation procedure and confidence interval are given in the Supplementary Material, Section S.3.2. The results suggest that the estimated reproduction number of the Tolima CL second outbreak was found to be 1.02 (see Table 6).

### 4.3. Comparison of Estimates of $R_{0}$ between Two Regions

The estimates of the basic reproduction number for Madrid outbreak was around three times more than the estimates of the reproduction number for Tolima outbreaks (see Table 7). The first outbreak of Tolima has larger reproduction number as compared to the corresponding number for the second outbreak.

## 5. Discussion

Proper surveillance is crucial for controlling leishmaniasis in endemic countries; however, there is a need to develop methods that can measure disease transmission rates effectively using existing limited data [29] and can be used to evaluate control programs [40]. Leishmaniasis-affected regions are primarily resource-constrained and hence face various challenges to gathering regular comprehensive data. In such scenarios, model-driven decisions might be helpful and can provide understanding of region-specific transmission dynamics [37]. In this study, we provide methodologies to estimate the basic reproduction number, *R*_0_, for CL with regional dependent factors. The estimation methods were tested using case studies from the two economically contrasting regions, Madrid, Spain and Tolima, Colombia. The Madrid model considers dog reservoir hosts (since most cases had contact with infected dogs) whereas the Tolima model takes into account the movement of military personnel on the transmission dynamics of CL. The three estimation procedures were developed for the two models to estimate their parameters using reported incidence data. Unlike the traditionally used estimating process, in which point estimation of model parameters is taken directly from independent studies reported in the literature, the methods in this research provide a simple but consistent way to estimate model parameters. The estimation of model parameters is followed by the estimation of the basic reproduction number, *R*_0_, and the computation of various epidemiologically important quantities, the type reproduction number (RT) vectorial capacity (VC) and entomological inoculation rates (EIR). 

Prior to the estimation of model parameters, outbreak-related CL incidence data from the two ecologically and epidemiologically different regions (Tolima, Colombia and Madrid, Spain) are first analyzed. Various differences are found in the outbreaks: (i) The Madrid incidence data were in months whereas the Tolima data were collected every week (this suggests a difference in the reporting systems of the two countries and potentially different infectious and latent periods between the regions), (ii) The outbreak in Madrid peaked in winters (Dec and Jan) as compared to the outbreak in Tolima, where the highest incidence was observed in Spring (April) and fall (in October), (iii) Each of the Tolima outbreaks was short lived (1/2 year) whereas the outbreak in Madrid lasted for 3 years (the Madrid outbreak was from 2009 to 2012 whereas the two Tolima outbreaks both occurred during 2016), (iv) there were two successive outbreaks in Tolima whereas there was a single outbreak in Madrid (the first outbreak in Tolima was much more lethal than its second outbreak), and (v) Dog reservoirs were important in the Madrid transmission cycle but in the Tolima outbreak, the frequently moving military population played a critical role in its spread.

The key parameter describing the spread of an infection is the basic reproduction numbers, *R*_0_, which is defined as the number of secondary infections generated by an infected index case in otherwise susceptible population. This study uses mathematical models to estimate *R*_0_ for the 2009–2012 CL outbreak in Madrid and the two CL outbreaks in Tolima during 2016. The mean estimates of *R*_0_ are found to be 3.1 for Madrid, 1.2 for the first outbreak of Tolima and 1.01 for the second outbreak of Tolima. The *R*_0_ estimate for Madrid seems to be significantly higher than corresponding estimate for Tolima. This could be a result of differences in the population density (60 persons/km^2^ in Tolima vs. 5400 persons/km^2^ in Madrid), climatic factors, human mobility, and/or health disparities in sub-communities [41]. In the Madrid outbreak, dogs were the main reservoir host, *P. perniciosus* was the principal vector of leishmania and *L. infantum* was the primary parasite species [5]. Epidemic outbreaks of CL in Tolima were caused by *L. braziliensis*, *L. guyanensis* and *L. panamensis*, with intra- and peri-domiciliary transmission. The military showed the highest incidence of CL due to the continuous deployment of troops to areas of high endemicity. 

Risk factors for CL such as urbanization, malnutrition, health seeking behaviors and disparity have been reported in the literature [42]; however, their impact on the dynamics of CL is less known [43]. This study attempts to provide a simple framework by which the impact of risk factors can be captured using limited reported data. We made some simplifying assumptions to reduce the dimension of the models and to obtain an explicit analytical formula for estimating *R*_0_. The data used here for fitting the model were obtained through passive case detection and therefore may be prone to high underreporting. Nevertheless, the results in this study suggest a *One Health* perspective, for example, if animals are key reservoirs of CL, interventions should not be human focused only, instead control programs should be heterogeneous, focusing on both human and animal hosts [44]. It suggests identification of disease-affected communities also in *Blue Marble Health* countries, wealthy nations with high GDP but also high endemicity of neglected diseases in hidden pockets [41]. Thus, the current estimation study needs to also include proper cost analysis in order to study transmission dynamics comprehensively [45]. As more data accumulate in the future, a more thorough analysis will allow for more accurate estimates of *R*_0_, together with less uncertainty around them and greater understanding of impact of socio-economic conditions on its estimates. 

## Figures and Tables

**Table 1:** Estimates of reproduction number (*R*_0_) from studies in the literature.

Study	Estm of *R*_0_	Model’s Feature	Data Used	Region; Disease
Dye et al. 1992 [17]	11	Dog hosts	Seroprevalence	Gozo island, Malta; CL
Reithinger et al. 2003 [22]	1.9	Dog hosts	Seroprevalence	Huánuco, Peru; CL
Bacaer et al. 2006 [23]	1.94	Seasonality in sandflies; intrinsic latent period distribution	Incidence	Chichaoua, Morocco; CL
Carlos Rosales et al. 2007 [24]	4.689 (Rio Blanco), 1.948 (Las Carmelitas)	Human hosts, canine hosts	Incidence	Rio Blanco & Las Carmelitas, Argentina; CL
Chaves 2018 [25]	1.64 (Constant mortality), 1.22 (smoothed variable mortality)	Dog hosts	Seroprevalence	Rural village, Panamá; CL
Biswas 2017 [26]	3.81	Human hosts	Incidence	South Sudan; VL
Costa et al. 2013 [27]	1.09 (low endemic), 1.29 (high endemic)	Dog hosts	Seroprevelence	Latin America; CL
Stauch et al. 2011 [28]	3.94	Human hosts	Incidence	Indian subcontinent; VL
Mubayi et al. 2010 [29]	1.3–2.1	Human hosts	Incidence	Bihar, India; VL

**Table 2:** Variables and parameters of model used for Madrid.

Symbol		Definition
*Parameters*		
*Reduced Model*	$s_{h}\left(0\right)$	Initial size of susceptible population
	β	Effective transmission rate
	$\gamma_{h}$	Per capita recovery rate
	$R_{0}^{2}$	Basic reproduction number
$\mu_{h}$		Per capita natural mortality rate in humans
$a_{h}$		Average number of bites received by a human
$\beta_{vh}$		Probability of transmission from vector to human in a bite
$\mu_{v}$		Per capita natural mortality rate in vectors
$b_{v}$		Average biting rate (avg. vector bites/(human or dog)/time
$\beta_{hv}$		Probability of transmission from human to vector in a bite
$\beta_{Av}$		Probability of transmission from dog to vector in a bite
$\mu_{A}$		Per capita natural mortality rate in dogs
$\beta_{vA}$		Probability of transmission from vector to dog in a bite
$\alpha_{A}$		Average number of bites received by a dog
*Variables*		
$X_{h}$		Number of susceptible humans
$Y_{h}$		Number infected humans
$Z_{h}$		Number of recovered humans
$X_{v}$		Density of susceptible vectors
$Y_{v}$		Density of infected vectors
$X_{A}$		Number of susceptible dogs
$Y_{A}$		Number of infected dogs

**Table 3:** Variables and parameters of the model used for Tolima, Colombia.

Symbol		Definition
*Parameters*		
*Reduced Model*	$s_{h}\left(0\right)$	Initial size of susceptible population
	β	Effective transmission rate
	$\gamma_{h}$	Per capita recovery rate
	$R_{0}^{2}$	Basic reproduction number
*Variables*		
$S_{v}$		Density of susceptible vectors
$i_{v}$		Density of infected vectors
$S_{c}$		Number of susceptible civilians
$i_{c}$		Number of infected civilians
$R_{c}$		Number of recovered civilians
$S_{m}$		Number of susceptible military individuals
$i_{m}$		Number of infected military individuals

**Table 4:** Model parameter estimates for Madrid-Spain.

Method	$s_{h}\left(0\right)$	$\beta_{eff}$	$\gamma_{h}$	$R_{0}^{2}$
EAC ^1^	711	0.000481	0.08593	3.9
EIC1 ^2^	629	0.000569	0.11699	3.1
EIC2 ^3^	606	0.000614	0.13117	2.8

^1^ Estimation using the observed curve of accumulated cases (EAC or Method 0), ^2^ Estimation using the observed incidence curve (EIC1 or Method 1), ^3^ Estimation using the observed incidence curve (EIC2 or Method 2).

**Table 5:** Model parameter estimates for Tolima, Colombia (first outbreak).

Method	$s_{c}\left(0\right)$	$\beta_{eff}$	$\gamma_{c}$	$R_{0}^{2}$
EAC ^1^	5,288	0.000272	1.13165	1.3
EIC1 ^2^	6,656	0.000266	1.47689	1.2
EIC2 ^3^	7,301	0.000272	1.69342	1.2

^1^ Estimation using the observed curve of accumulated cases (EAC or M0), ^2^ Estimation using the observed incidence curve (EIC1 or M1), ^3^ Estimation using the observed incidence curve (EIC2 or M2).

**Table 6:** Model parameter estimates for Tolima, Colombia (second outbreak).

Method	$s_{c}\left(0\right)$	$\beta_{eff}$	$\gamma_{c}$	$R_{0}^{2}$
EAC ^1^	41,031	0.000412	16.59690	1.019
EIC1 ^2^	38,706	0.000382	14.47438	1.021
EIC2 ^3^	44,325	0.000384	16.71430	1.018

^1^ Estimation using the observed curve of accumulated cases (EAC or M0), ^2^ Estimation using the observed incidence curve (EIC or M1), ^3^ Estimation using the observed incidence curve (EIC or M2).

**Table 7:** Comparison of estimates of the basic reproduction number for the outbreaks.

Madrid, Spain:	
Using accumulated cases (Method 0)	$R_{0}^{2}=3.9$
Using incidence derivative (Method 1)	$R_{0}^{2}=3.1$
Using discrete incidence (Method 2)	$R_{0}^{2}=2.9$
Tolima, Colombia (first outbreak)	
Using accumulated cases (Method 0)	$R_{0}^{2}=1.3$
Using incidence derivative (Method 1)	$R_{0}^{2}=1.2$
Using discrete incidence (Method 2)	$R_{0}^{2}=1.2$
Tolima, Colombia (second outbreak)	
using accumulated cases (method 0)	$R_{0}^{2}=1.01$
using incidence derivative (method 1)	$R_{0}^{2}=1.02$
Using discrete incidence (Method 2)	$R_{0}^{2}=1.02$

## Supplementary Materials

The following are available online at http://www.mdpi.com/2414-6366/3/2/43/s1, Figure S1: Cumulative cases of leishmaniasis in the Madrid from July 2009 to March 2012, Figure S2: Cumulative cases of leishmaniasis in Tolima-Colombia during 2016, Figure S3: Flow chart representing the second mathematical model for Tolima.
