# Supplementary material for: A Comparative Assessment of Epidemiologically Different Cutaneous Leishmaniasis Outbreaks in Madrid, Spain and Tolima, Colombia: An Estimation of the Reproduction Number via a Mathematical Model
Source: Trop Med Infect Dis. 2018 Apr 19;3(2):43. doi: 10.3390/tropicalmed3020043 (PMC6073504; doi:10.3390/tropicalmed3020043)
Supplement: Supplementary file 1 [file tropicalmed-03-00043-s001.pdf]

Tropical Medicine and Infectious Disease

MDPI

A Comparative Assessment of Epidemiologically Different Cutaneous Leishmaniasis Outbreaks in Madrid-Spain and Tolima-Colombia: An Estimation of the Reproduction Number via a Mathematical Model

Anuj Mubayi [1,*], Marlio Paredes [1,2,3,4] and Juan Ospina [5]

Supplementary Material

S.1. Observed curve of accumulated cases

The observed curve of accumulated cases for Madrid outbreak is shown in the following figure

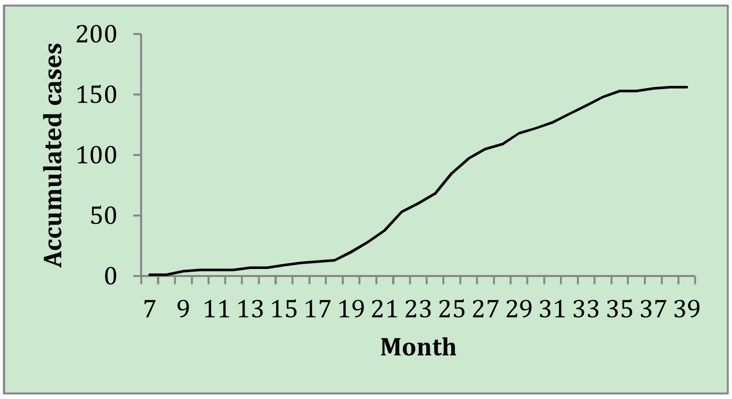

Figure S1. Cumulative cases of Leishmaniasis in the Madrid from July 2009 to March 2012 [**Error! Reference source not found.**].

The observed curve of accumulated cases starts from the month 7 (July 2009) and ends at the month 39 (March 2012). The total number of cases was 266.

The observed curve of accumulated cases for the two Tolima outbreaks is shown in the following figure.

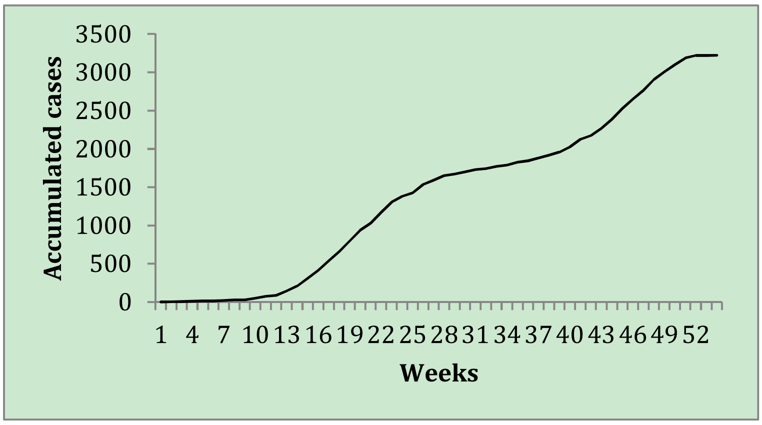

Figure S2. Cumulative cases of Leishmaniasis in Tolima-Colombia during 2016 [**Error! Reference source not found.**].

The observed curve of accumulated cases starts from the epidemiological week 1 (first week of January 2016) and ends at the epidemiological week 52 (first week of January 2017). The total number of cases was 3223. We observe clearly that two outbreaks held during 2016 at Tolima, Colombia. The first outbreak occurred since week 1 to week 35 and the second outbreak occurred since week 36 to week 52.

The differences in some of factors that may impact health disparity between Madrid-Spain and Tolima-Colombia are shown in following table.

	**Madrid-Spain (2009-2012) [2008 Statistics]**	**Tolima-Colombia (2016) [2015 Statistics]**
**GDP Per Capita**	35,578 USD	6044 USD
**Population Size**	3.2 M (2017)	1.41 M (2016)
**Avg. Annual Temperature**	15 °C (1981-2010)	24°C
**Avg. Annual Precipitation**	421.5mm (1981-2010)	1500 mm
**Population Density**	5265,91 hab./km^2 (2017)	59,94 hab/km^2 (2016)
**Proportion Below Poverty Line**	15.6 %	32.9 %
**Gini Coefficient**	36.0 (2014 World Bank)	51.1 (2015 World Bank)
**Mean Income per Unit of Consumption**	Euro 17,800	$8,304 (per year Colombia)
**Unemployment rate**	13.8% (2017)	13% (2017)

S.2. Mathematical Models

S.2.1. Mathematical Model for Leishmaniasis in Madrid-Spain

We use a model proposed in [**Error! Reference source not found.**] which has the form

$$\frac{dX_h}{dt} = \mu_h N_h - \frac{a_h \beta_{vh} X_h Y_v}{N_h + N_A} - \mu_h X_h \qquad (S1)$$

$$\frac{dY_h}{dt} = \frac{a_h \beta_{vh} X_h Y_v}{N_h + N_A} - \gamma_h Y_h - \mu_h Y_h \qquad (S2)$$

$$\frac{dZ_h}{dt} = \gamma_h Y_h - \mu_h Z_h \qquad (S3)$$

$$\frac{dX_v}{dt} = \Lambda - \frac{b_v \beta_{hv} X_v Y_h}{N_h + N_A} - \frac{b_v \beta_{Av} X_v Y_A}{N_h + N_A} - \mu_v X_v \qquad (S4)$$

$$\frac{dY_v}{dt} = \frac{b_v \beta_{hv} X_v Y_h}{N_h + N_A} + \frac{b_v \beta_{Av} X_v Y_A}{N_h + N_A} - \mu_v Y_v \qquad (S5)$$

$$\frac{dX_A}{dt} = \mu_A N_A - \frac{\alpha_A \beta_{vA} X_A Y_v}{N_h + N_A} - \mu_A X_A \qquad (S6)$$

$$\frac{dY_A}{dt} = \frac{\alpha_A \beta_{vA} X_A Y_v}{N_h + N_A} - \mu_A Y_A \qquad (S7)$$

The equations $(S1)$, $(S2)$ and $(S3)$ are for human individuals, the equations $(S4)$ and $(S5)$ are for vectors; and equations $(S6)$ and $(S7)$ are for the animal reservoir. The parameters in the equations are the usual in an extended SIR model and their detailed meanings can be founded in [**Error! Reference source not found.**]. The basic reproductive number for the model $(S1)$-$(S7)$ has the form

$$R_0^2 = \frac{\beta_{hv} b_v N_h}{(N_h + N_A)(\mu_h + \gamma)} \frac{\beta_{vh} a_h \Lambda/\mu_v}{(N_h + N_A)\mu_v} + \frac{\beta_{Av} b_v N_A}{(N_h + N_A)\mu_A} \frac{\beta_{vA} \alpha_A \Lambda/\mu_v}{(N_h + N_A)\mu_v} \quad (S8)$$

At a first glance it seems the model ($S1$)-($S7$) is very complex to try to fix the curve of accumulated cases. Then we use a strategy also applied in [**Error! Reference source not found.**] and inspired in [**Error! Reference source not found.**].

Now, we transform the complex model ($S1$)-($S7$) into an effective simple SIR model. Through this transformation the equation ($S8$) will be derived again. Assuming the number of infected reservoir animals are constant during the outbreak and $X_A(t) = N_A$, the equation ($S7$) is reduced to

$$\frac{\alpha_A \beta_{vA} N_A Y_v}{N_h + N_A} - \mu_A Y_A = 0 \quad (S9)$$

and from this equation we obtain

$$Y_A = \frac{\alpha_A \beta_{vA} N_A Y_v}{(N_h + N_A)\mu_A} \quad (S10)$$

On the other hand, assuming that the number of infected vectors are keeping approximately constant during the outbreak and $X_v(t) = N_v$; the equation ($S5$) is reduced to

$$\frac{b_v \beta_{hv} X_v Y_h}{N_h + N_A} + \frac{b_v \beta_{Av} X_v Y_A}{N_h + N_A} = \mu_v Y_v \quad (S11)$$

From ($S10$) and ($S11$) we have

$$Y_v = -\frac{b_v \beta_{hv} N_v Y_h (N_h + N_A)\mu_A}{-\mu_A \mu_v N_h^2 - 2\mu_A \mu_v N_h N_A - \mu_A \mu_v N_A^2 + b_v \beta_{Av} N_v \alpha_A \beta_{vA} N_A} \quad (S12)$$

Then, substituting ($S12$) in ($S1$) we obtain

$$\frac{dX_h}{dt} = \mu_h N_h + \frac{a_h \beta_{vh} X_h b_v \beta_{hv} N_v Y_h \mu_A}{-\mu_A \mu_v N_h^2 - 2\mu_A \mu_v N_h N_A - \mu_A \mu_v N_A^2 + b_v \beta_{Av} N_v \alpha_A \beta_{vA} N_A} \quad (S13)$$

and with the approximation $X_h(t) = N_h$, the equation ($S13$) takes the form

$$\frac{dX_h}{dt} = -\frac{a_h \beta_{vh} X_h b_v \beta_{hv} N_v Y_h \mu_A}{\mu_A \mu_v N_h^2 + 2\mu_A \mu_v N_h N_A + \mu_A \mu_v N_A^2 - b_v \beta_{Av} N_v \alpha_A \beta_{vA} N_A} \quad (S14)$$

Now, substituting ($S12$) in ($S2$) we obtain

$$\frac{dY_h}{dt} = -\frac{a_h \beta_{vh} X_h b_v \beta_{hv} N_v Y_h \mu_A}{-\mu_A \mu_v N_h^2 - 2\mu_A \mu_v N_h N_A - \mu_A \mu_v N_A^2 + b_v \beta_{Av} N_v \alpha_A \beta_{vA} N_A} - \gamma_h Y_h - \mu_h Y_h \quad (S15)$$

Observing ($S14$) and ($S15$) it is possible to define the following effective infectiousness

$$\beta_{eff} = \frac{a_h \beta_{vh} b_v \beta_{hv} N_v \mu_A}{\mu_A \mu_v N_h^2 + 2\mu_A \mu_v N_h N_A + \mu_A \mu_v N_A^2 - b_v \beta_{Av} N_v \alpha_a \beta_{vA} N_A} \quad (S16)$$

Using this, the equations ($S14$) and ($S15$) are reduced to

$$\frac{dX_h}{dt} = -\beta_{eff} X_h Y_h \quad (S17)$$

$$\frac{dY_h}{dt} = \beta_{eff} X_h Y_h - \gamma_h Y_h - \mu_h Y_h \quad (S18)$$

Looking at ($S18$) and ($S3$) we observe that it is possible to define an effective removal constant given by

$$\gamma_{eff,h} = \gamma_h + \mu_h \quad (S19)$$

Now, using ($S19$) we can rewrite equations ($S18$) and **Error! Reference source not found.** in the following way

$$\frac{dY_h}{dt} = \beta_{eff} X_h Y_h - \gamma_{eff,h} Y_h \quad (S20)$$

$$\frac{dZ_h}{dt} = \gamma_{eff,h} Y_h \qquad (S21)$$

Hence, equations ($S17$), ($S20$) and ($S21$) give us an effective simple SIR model for leishmaniasis. Now, from ($S15$) we can obtain the condition for the existence of an outbreak

$$-\frac{a_h \beta_{vh} X_h b_v \beta_{hv} N_v Y_h \mu_A}{-\mu_A \mu_v N_h^2 - 2\mu_A \mu_v N_h N_A - \mu_A \mu_v N_A^2 + b_v \beta_{Av} N_v \alpha_A \beta_{vA} N_A} - \gamma_h Y_h - \mu_h Y_h > 0$$

and from here we derive the threshold condition

$$a_h \beta_{vh} X_h b_v \beta_{hv} N_v \mu_A - \gamma_h \mu_A \mu_v N_h^2 - 2\gamma_h \mu_A \mu_v N_h N_A - \gamma_h \mu_A \mu_v N_A^2 + \gamma_h b_v \beta_{Av} N_v \alpha_A \beta_{vA} N_A - \mu_h \mu_A \mu_v N_h^2 - 2\mu_h \mu_A \mu_v N_h N_A - \mu_h \mu_A \mu_v N_A^2 + \mu_h b_v \beta_{Av} N_v \alpha_A \beta_{vA} N_A > 0 \qquad (S22)$$

The threshold condition is rewritten as

$$R_0 > 1 \qquad (S23)$$

where

$$R_0^2 = \frac{a_h \beta_{vh} N_h \beta_{hv} b_v N_v}{\mu_v (N_h + N_A)^2 (\gamma_h + \mu_h)} + \frac{b_v N_v N_A \beta_{Av} \alpha_A \beta_{vA}}{\mu_A \mu_v (N_h + N_A)^2} \qquad (S24)$$

S.2.2. Mathematical Model for Leishmaniasis in Tolima-Colombia

We use a model proposed in [**Error! Reference source not found.**] which has the form

$$\frac{dS_v}{dt} = \Lambda_v - \frac{b\beta S_v i_m}{N_c + N_m} - \frac{b\beta S_v i_c}{N_c + N_m} - \mu_v S_v \qquad (S25)$$

$$\frac{di_v}{dt} = \frac{b\beta S_v i_m}{N_c + N_m} + \frac{b\beta S_v i_c}{N_c + N_m} - \mu_v i_v \qquad (S26)$$

$$\frac{dS_c}{dt} = \Lambda_c - \frac{b\beta S_c i_v}{N_v} - \mu_c S_c \qquad (S27)$$

$$\frac{di_c}{dt} = \frac{b\beta S_c i_v}{N_v} - \mu_c i_c - \gamma_c i_c \qquad (S28)$$

$$\frac{dR_c}{dt} = -\mu_c R_c + \gamma_c i_c \qquad (S29)$$

$$\frac{dS_m}{dt} = (1-q)\Lambda_m - \frac{b\beta S_m i_v}{N_v} - \mu_m S_m \qquad (S30)$$

$$\frac{di_m}{dt} = q\Lambda_m + \frac{b\beta S_m i_v}{N_v} - \mu_m i_m \qquad (S31)$$

The equations ($S25$) and ($S26$) are for vectors (*Phlebotomus*), the equations ($S27$), ($S28$) and ($S29$) are for civilian individuals; and the equations ($S30$) and ($S31$) are for the military individuals. The parameters in the equations are the usual in an extended SIR model and their detailed meaning can be founded in [**Error! Reference source not found.**]. According to this model the military individuals are penetrating into the deep jungle and then carry on the disease to the urban zones where the civilian people are susceptible to the disease transmitted by the *Phlebotomus*. The basic reproduction number for the model has the form [**Error! Reference source not found.**]

$$R_0^2 = \frac{b^2 \beta^2 \Lambda_v \Lambda_m}{N_v \mu_v^2 \mu_m (N_c + N_m)} + \frac{b^2 \beta^2 \Lambda_v \Lambda_c}{N_v \mu_v^2 \mu_c (N_c + N_m)(\mu_c + \gamma_c)} \qquad (S32)$$

At a first glance it seems the model $(S25)$ - $(S31)$ is very complex in order to fix the curve of accumulated cases. Then we use a strategy also applied in [**Error! Reference source not found.**] and inspired in [**Error! Reference source not found.**].

We transform the complex model $(S25)$ - $(S31)$ into an effective simple SIR model. Doing such transformation, the equation $(S32)$ will be derived again. We assume that the number of infected military individuals is constant during the outbreak and $S_m(t) = N_m$ in equation $(S31)$, also assuming that the number of infected vectors is keeping approximately constant during the outbreak and $S_v(t) = N_v$ in equation $(S26)$. Then we deduce that

$$i_v = -\frac{N_v b\beta(\mu_m i_c + \gamma_m i_c + q\Lambda_m)}{b^2\beta^2 \mathrm{N}_m - \mu_v N_c \mu_m - \mu_v N_c \gamma_m - \mu_v N_m \mu_m - \mu_v N_m \gamma_m} \quad (S33)$$

Replacing $(S33)$ in $(S27)$ we obtain

$$\frac{dS_c}{dt} = \Lambda_c + \frac{b^2\beta^2 \mathrm{S}_c(\mu_m i_c + \gamma_m i_c + q\Lambda_m)}{b^2\beta^2 \mathrm{N}_m - \mu_v N_c \mu_m - \mu_v N_c \gamma_m - \mu_v N_m \mu_m - \mu_v N_m \gamma_m} - \mu_c S_c$$

and with the approximation $\Lambda_c = \mu_c S_c$ and $q = 0$, this equation (39) takes the form

$$\frac{dS_c}{dt} = \frac{b^2\beta^2 \mathrm{S}_c i_c(\mu_m + \gamma_m)}{b^2\beta^2 \mathrm{N}_m - \mu_v N_c \mu_m - \mu_v N_c \gamma_m - \mu_v N_m \mu_m - \mu_v N_m \gamma_m} \quad (S34)$$

Now, if we substitute $(S33)$ in $(S28)$ and taking $q = 0$ we obtain

$$\frac{di_c}{dt} = -\frac{b^2\beta^2 \mathrm{S}_c i_c(\mu_m + \gamma_m)}{b^2\beta^2 \mathrm{N}_m - \mu_v N_c \mu_m - \mu_v N_c \gamma_m - \mu_v N_m \mu_m - \mu_v N_m \gamma_m} - \mu_c i_c - \gamma_c i_c \quad (S35)$$

From these two last equations we can define the following effective infectiousness

$$\beta_{eff} = -\frac{b^2\beta^2(\mu_m + \gamma_m)}{b^2\beta^2 N_m - \mu_v N_c \mu_m - \mu_v N_c \gamma_m - \mu_v N_m \mu_m - \mu_v N_m \gamma_m} \quad (S36)$$

Using $(S36)$, we rewrite the equations $(S34)$ and $(S35)$ in the following way

$$\frac{dS_c}{dt} = -\beta_{eff} \mathrm{S}_c i_c \quad (S37)$$

$$\frac{di_c}{dt} = \beta_{eff} \mathrm{S}_c i_c - \gamma_{c,eff} i_c \quad (S38)$$

where $\gamma_{c,eff} = \mu_c + \gamma_c$. With all these approximations the equation $(S29)$ is rewritten as

$$\frac{dR_c}{dt} = \gamma_{c,eff} i_c \quad (S39)$$

Then, we observe that the equations $(S37)$, $(S38)$ and $(S39)$ give us an effective simple SIR model for leishmaniasis.

Now, from $(S35)$ the condition for the existence of an outbreak is

$$-\frac{b^2\beta^2 \mathrm{S}_c i_c(\mu_m + \gamma_m)}{b^2\beta^2 N_m - \mu_v N_c \mu_m - \mu_v N_c \gamma_m - \mu_v N_m \mu_m - \mu_v N_m \gamma_m} - \mu_c i_c - \gamma_c i_c > 0$$

Then, from here we derive the threshold condition

$$b^2\beta^2 \mathrm{S}_c \mu_m + b^2\beta^2 \mathrm{S}_c \gamma_m + \mu_c b^2\beta^2 N_m - \mu_c \mu_v N_c \mu_m - \mu_c \mu_v N_c \gamma_m - \mu_c \mu_v N_m \mu_m - \mu_c \mu_v N_m \gamma_m + \gamma_c b^2\beta^2 N_m - \gamma_c \mu_v N_c \mu_m - \gamma_c \mu_v N_c \gamma_m - \gamma_c \mu_v N_m \mu_m - \gamma_c \mu_v N_m \gamma_m > 0 \quad (S40)$$

The threshold condition is rewritten as

$$R_0 > 1 \quad (S41)$$

where

$$R_0^2 = \frac{b^2\beta^2 N_c}{\mu_v(\mu_c + \gamma_c)(N_c + N_m)} + \frac{b^2\beta^2 N_m}{\mu_v(\mu_m + \gamma_m)(N_c + N_m)} \quad (S42)$$

S.2.3. Second Mathematical Model for Leishmaniasis in Tolima-Colombia

In Tolima case, we also can consider the following model

$$\frac{dS_v}{dt} = \Lambda_v - \frac{b\beta S_v i_m}{N_c + N_m} - \frac{b\beta S_v i_c}{N_c + N_m} - \mu_v S_v \qquad (S43)$$

$$\frac{di_v}{dt} = \frac{b\beta S_v i_m}{N_c + N_m} + \frac{b\beta S_v i_c}{N_c + N_m} - \mu_v i_v \qquad (S44)$$

$$\frac{dS_c}{dt} = \Lambda_c - \frac{b\beta S_c i_v}{N_v} - \mu_c S_c - m_1 S_c + m_2 S_m \qquad (S45)$$

$$\frac{di_c}{dt} = \frac{b\beta S_c i_v}{N_v} - \mu_c i_c - \gamma_c i_c \qquad (S46)$$

$$\frac{dR_c}{dt} = -\mu_c R_c + \gamma_c i_c + m_2 R_m - m_1 R_c \qquad (S47)$$

$$\frac{dS_m}{dt} = \Lambda_a - \frac{b\beta S_m i_v}{N_v} - \mu_m S_m + m_1 S_c - m_2 S_m \qquad (S48)$$

$$\frac{di_m}{dt} = \Lambda_b + \frac{b\beta S_m i_v}{N_v} - \mu_m i_m - \gamma_m i_m \qquad (S49)$$

$$\frac{dR_m}{dt} = -\mu_m R_m + \gamma_m i_m - m_2 R_m + m_1 R_c \qquad (S50)$$

The equations ($S59$)for vectors (*Phlebotomus*), equations ($S27$) are for civilian individuals; equations ($S30$) are for military individuals. According to this model, the military individuals move between jungle and urban zones (Λ_a and Λ_b; the movement of troops between modeled region and other areas is also captured in these constants). The individuals can be recruited into military from civilians as rate m_1 and can leave military population at the rate m_2. The following figure contains flow-chart of the model, it includes recover compartment for military population and is taking account mobility between military and civilian populations.

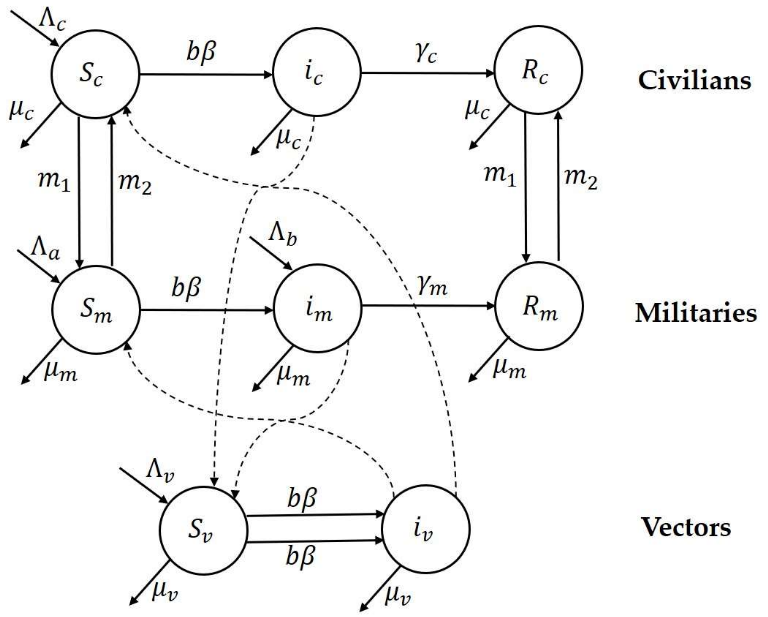

Figure S3. Flow chart representing the second mathematical model for Tolima.

It is easy to see that the equilibrium point of our system ($S25$) is

$$E^* = (S_v^*, i_v^*, S_c^*, i_c^*, R_c^*, S_m^*, i_m^*, R_m^*) = \left(\frac{\Lambda_v}{\mu_v}, 0, S_c^*, 0,0, S_m^*, 0,0\right) \quad (S51)$$

where

$$S_c^* = \frac{(\mu_m + m_2)\Lambda_c + m_2\Lambda_m}{(\mu_m + m_2)(\mu_c + m_1) - m_1 m_2} \quad (S52)$$

and

$$S_m^* = \frac{(\mu_c + m_1)\Lambda_m + m_1\Lambda_c}{(\mu_m + m_2)(\mu_c + m_1) - m_1 m_2} \quad (S53)$$

We use the new generation matrix approach to calculate the basic reproduction number for our system ($S25$). Then, the vector of new infection rates is

$$\mathcal{F} = \begin{pmatrix} \dfrac{b\beta S_v i_m}{N_c + N_m} + \dfrac{b\beta S_v i_c}{N_c + N_m} \\ \dfrac{b\beta S_v i_v}{N_v} \\ \dfrac{b\beta S_m i_v}{N_v} \end{pmatrix} \quad (S54)$$

and the vector of all other rates is

$$\mathcal{V} = \begin{pmatrix} \mu_v i_v \\ (\mu_c + \gamma_c) i_c \\ (\mu_m + \gamma_m) i_m \end{pmatrix} \quad (S55)$$

Then, the next generation matrix is

$$B = FV^{-1} = D\mathcal{F}(E^*)D\mathcal{V}(E^*) = \begin{pmatrix} 0 & \dfrac{b\beta S_v^*}{(N_c + N_m)(\mu_c + \gamma_c)} & \dfrac{b\beta S_v^*}{(N_c + N_m)(\mu_m + \gamma_m)} \\ \dfrac{b\beta S_c^*}{\mu_v N_v} & 0 & 0 \\ \dfrac{b\beta S_m^*}{\mu_v N_v} & 0 & 0 \end{pmatrix} \quad (S56)$$

and we obtain the basic reproductive as

$$R_0^2 = \frac{b^2\beta^2 S_v^* S_c^*}{\mu_v N_v (N_c + N_m)(\mu_c + \gamma_c)} + \frac{b^2\beta^2 S_v^* S_m^*}{\mu_v N_v (N_c + N_m)(\mu_m + \gamma_m)} \quad (S57)$$

or

$$R_0^2 = \frac{b^2\beta^2 \Lambda_v \left((\mu_m + m_2)\Lambda_c + m_2\Lambda_a\right)}{\mu_v^2 N_v (N_c + N_m)(\mu_c + \gamma_c)\left((\mu_m + m_2)(\mu_c + m_1) - m_1 m_2\right)} + \frac{b^2\beta^2 \Lambda_v \left((\mu_c + m_1)\Lambda_a + m_1\Lambda_c\right)}{\mu_v^2 N_v (N_c + N_m)(\mu_c + \gamma_c)\left((\mu_m + m_2)(\mu_c + m_1) - m_1 m_2\right)} \quad (S58)$$

S.3. Parameter estimation procedure

S.3.1. Spain case

The effective SIR model ($S17$), ($S20$) and ($S21$) can be solved approximately as

$$R(t) = \frac{\rho^2\left(\frac{s}{\rho} - 1 - \alpha \tanh(-0.5\alpha\gamma_h t + \phi)\right)}{s} \qquad (S59)$$

where $s = N_h$; and

$$\alpha = \sqrt{\left(\frac{s}{\rho} - 1\right)^2 + \frac{2s}{\rho^2}} \qquad (S60)$$

$$\phi = \frac{1}{2}\ln\left(\frac{\alpha\rho + s - \rho}{\alpha\rho - s + \rho}\right) \qquad (S61)$$

$$\rho = \frac{\gamma_h}{\beta} \qquad (S62)$$

with $R(t) = Z_h(t)$, $\beta = \beta_{eff}$ and $\gamma_h = \gamma_{eff,h}$.

Estimation using the observed curve of accumulated cases

Now, we use $(S59)$ to fix the observed curve of accumulated cases. In section *S.4.* is included the corresponding code to obtain the following results

Descriptive Statistics for Variables					
Variable	Minimum value	Maximum value	Mean value	Standard dev.	
Mes	7	39	23	9.66954	
TC	1	266	109.8182	98.22438	
Calculated Parameter Values					
Parameter	Initial guess	Final estimate	Standard error	t	Prob(t)
Gamma	1E-005	0.0859279745	0.01518749	5.66	0.00001
Beta	1E-007	0.000480881087	5.542945E-005	8.68	0.00001
s	1E+006	711.877322	68.45965	10.40	0.00001
Analysis of Variance					
Source	DF	Sum of Squares	Mean Square	F value	Prob(F)
Regression	2	307892	153946	5465.86	0.00001
Error	30	844.9504	28.16501		
Total	32	308736.9			
90.000% Confidence Intervals					
Parameter	Lower limit	Best estimate	Upper limit		
gamma	0.0601512357	0.0859279745	0.111704713		
beta	0.000386804281	0.000480881087	0.000574957893		
s	595.685198	711.877322	828.069447		

The best estimates for the parameters are

$s_h = 711.877322$ $\beta_{eff} = 0.000480881087$ $\gamma_{eff,h} = 0.0859279745$

Using such values, we obtain that $R_0^2 = 3.983898636$.

Estimation using the observed incidence curve (Method 1)

Taking the temporal derivative of $(S59)$ we obtain the theoretical incidence curve given by

$$\frac{dR}{dt}(t) = \frac{1}{2}\frac{\rho^2\alpha^2\gamma}{s\cosh\left(-\frac{\alpha\gamma}{2}t + \phi\right)^2} \qquad (S63)$$

and we use this to fix the observed curve of incidence. In section *S.4.* is included the corresponding code to obtain the following results

Descriptive Statistics for Variables					
Variable	Minimum value	Maximum value	Mean value	Standard dev.	
Mes	7	39	23	9.66954	
INC	0	31	8.060606	6.576185	
Calculated Parameter Values					
Parameter	Initial guess	Final estimate	Standard error	t	Prob(t)
Gamma	1E-010	0.116995258	0.07885037	1.48	0.14830
Beta	1E-009	0.000569267647	0.000206206	2.76	0.00974
s	1900	629.288339	139.822	4.50	0.00010
Analysis of Variance					
Source	DF	Sum of Squares	Mean Square	F value	Prob(F)
Regression	2	954.631	477.3155	33.36	0.00001
Error	30	429.2478	14.30826		
Total	32	1383.879			
95.000% Confidence Intervals					
Parameter	Lower limit	Best estimate	Upper limit		
gamma	-0.0440382383	0.116995258	0.278028755		
beta	0.000148139901	0.000569267647	0.000990395392		
s	343.734575	629.288339	914.842104		

The best estimates for the parameters are

$s_h = 629.288339 \qquad \beta_{eff} = 0.000569267647 \qquad \gamma_{eff,h} = 0.116995258$

Using these values, we obtain $R_0^2 = 3.061948818$.

<u>Estimation using the observed curve of accumulated cases (Method 2)</u>

Other method to obtain the theoretical incidence is to use $(S59)$ two successive times and take the difference between such two times, namely

$$\mathrm{Inc}(t) = \frac{\rho^2\left(\frac{s}{\rho} - 1 - \alpha \tanh\left(-\frac{\alpha\gamma}{2}t + \phi\right)\right)}{s} - \frac{\rho^2\left(\frac{s}{\rho} - 1 - \alpha \tanh\left(-\frac{\alpha\gamma}{2}(t-1) + \phi\right)\right)}{s} \qquad (S64)$$

This equation can be simplified as

$$\mathrm{Inc}(t) = \frac{\rho^2\alpha\left(\tanh\left(-\frac{\alpha\gamma}{2}t + \phi\right) - \tanh\left(-\frac{\alpha\gamma}{2}t + \frac{\alpha\gamma}{2} + \phi\right)\right)}{s} \qquad (S65)$$

We use $(S65)$ to fix the observed curve of incidence. In section *S.4.* is included the corresponding code to obtain the following results

Descriptive Statistics for Variables					
Variable	Minimum value	Maximum value	Mean value	Standard dev.	
Mes	7	39	23	9.66954	
TC	0	31	8.060606	6.576185	
Calculated Parameter Values					
Parameter	Initial guess	Final estimate	Standard error	t	Prob(t)
Gamma	1E-005	0.131176236	0.0869062	1.51	0.14166
Beta	1E-014	0.000614420024	0.0002060138	2.98	0.00563
s	6000	606.396847	109.9455	5.52	0.00001
Analysis of Variance					

Source	DF	Sum of Squares	Mean Square	F value	Prob(F)
Regression	2	954.6061	477.3155	33.36	0.00001
Error	30	429.2727	14.30909		
Total	32	1383.879			
95.000% Confidence Intervals					
Parameter	Lower limit	Best estimate	Upper limit		
gamma	-0.0463094127	0.131176236	0.308661885		
beta	0.00019368491	0.000614420024	0.00103515514		
s	381.858844	606.396847	830.934849		

The best estimates for the parameters are

$s_h = 606.396847$ $\beta_{eff} = 0.000614420024$ $\gamma_{eff,h} = 0.131176236$

Using these values, we obtain $R_0^2 = 2.840319075$.

S.3.2. Colombia case

The effective SIR model for Tolima-Colombia ($S37$), ($S38$) and ($S39$) has essentially the same solution of the effective SIR model for Madrid-Spain, given by equations ($S59$)-($S62$) with $s = N_c$, $R(t) = R_c(t)$, $\beta = \beta_{eff}$ and $\gamma_h = \gamma_{c,eff}$. Then, we use again ($S59$) to fix the observed curve of accumulated cases for each outbreak in Tolima-Colombia. Section *S.5.* contains the corresponding code to obtain the following results,

Estimation using the observed curve of accumulated cases

First outbreak

Descriptive Statistics for Variables					
Variable	Minimum value	Maximum value	Mean value	Standard dev.	
Mes	1	35	18	10.24695	
TC	1	1825	790.1429	723.2331	
Calculated Parameter Values					
Parameter	Initial guess	Final estimate	Standard error	t	Prob(t)
Gamma	1E-005	1.13165588	0	1.0E+030	0.00001
Beta	1E-007	0.000272524001	0	1.0E+030	0.00001
s	1E+006	5288.63267	0	1.0E+030	0.00001
Analysis of Variance					
Source	DF	Sum of Squares	Mean Square	F value	Prob(F)
Regression	2	1.776487E+007	8882433	14665.54	0.00001
Error	32	19381.34	605.6669		
Total	34	1.778425E+007			

Unable to compute confidence intervals because the covariance matrix could not be computed.

The best estimates for the parameters are

$s_c = 5288.63267,$ $\beta_{eff} = 0.000272524001,$ $\gamma_{c,eff} = 1.13165588.$

Using such values, we obtain that $R_0^2 = 1.273602126$.

Second outbreak

Descriptive Statistics for Variables					
Variable	Minimum value	Maximum value	Mean value	Standard dev.	
Mes	1	19	10	5.627314	
TC	20	1398	721.4211	518.8136	

Calculated Parameter Values					
Parameter	Initial guess	Final estimate	Standard error	t	Prob(t)
Gamma	1E-005	16.5969	16.5969	0	0.00001
Beta	1E-007	0.000412022367	0	1.0E+030	0.00001
s	1000	1E-007	0.000412022367	1.0E+030	0.00001
Analysis of Variance					
Source	DF	Sum of Squares	Mean Square	F value	Prob(F)
Regression	2	4836994	2418497	4823.58	0.00001
Error	16	8022.241	501.3901		
Total	318	4845017			

Unable to compute confidence intervals because the covariance matrix could not be computed.

The best estimates for the parameters are

$s_c = 41031.1717,$ $\beta_{eff} = 0.000412022367,$ $\gamma_{c,eff} = 16.5969.$

Using such values, we obtain that $R_0^2 = 1.018609528$.

Estimation using the observed incidence curve (Method 1)

Here we use ($S63$) to fix the observed curves of incidence for the two outbreaks. In section *S.5.* is included the corresponding code to obtain the following results.

First outbreak

Descriptive Statistics for Variables					
Variable	Minimum value	Maximum value	Mean value	Standard dev.	
Mes	1	35	18	10.24695	
TC	0	145	52.11429	48.82669	
Calculated Parameter Values					
Parameter	Initial guess	Final estimate	Standard error	t	Prob(t)
Gamma	1E-010	1.4768931	0	1.0E+030	0.00001
Beta	1E-009	0.000265678781	0	1.0E+030	0.00001
s	1900	6656.64572	0	1.0E+030	0.00001
Analysis of Variance					
Source	DF	Sum of Squares	Mean Square	F value	Prob(F)
Regression	2	70974.4	35487.2	112.62	0.00001
Error	32	10083.15	315.0983		
Total	34	81057.54			

Unable to compute confidence intervals because the covariance matrix could not be computed.

The best estimates for the parameters are

$s_c = 6656.64572,$ $\beta_{eff} = 0.000265678781,$ $\gamma_{c,eff} = 1.4768931.$

Using such values, we obtain that $R_0^2 = 1.197466167$.

Second outbreak

Descriptive Statistics for Variables					
Variable	Minimum value	Maximum value	Mean value	Standard dev.	
Mes	1	17	9	5.049752	
TC	20	142	82	40.02031	

Calculated Parameter Values					
Parameter	Initial guess	Final estimate	Standard error	t	Prob(t)
Gamma	1E-010	14.4743765	0	1.0E+030	0.00001
Beta	1E-009	14.4743765	0	1.0E+030	0.00001
s	1900	38706.6128	0	1.0E+030	0.00001
Analysis of Variance					
Source	DF	Sum of Squares	Mean Square	F value	Prob(F)
Regression	2	20965.65	10482.82	31.49	0.00001
Error	14	31.49	332.8822		
Total	16	25626			

Unable to compute confidence intervals because the covariance matrix could not be computed.

The best estimates for the parameters are

$s_c = 38706.6128, \quad \beta_{eff} = 0.000381686495, \quad \gamma_{c,eff} = 14.4743765.$

Using such values, we obtain that $R_0^2 = 1.020685856$.

Estimation using the observed curve of accumulated cases (Method 2)

We use ($S65$) to fix the observed curve of incidence for the two outbreaks. Section *S.5.* contains the corresponding code to obtain the following results.

First outbreak

Descriptive Statistics for Variables					
Variable	Minimum value	Maximum value	Mean value	Standard dev.	
Mes	1	35	18	10.24695	
TC	0	145	52.11429	48.82669	
Calculated Parameter Values					
Parameter	Initial guess	Final estimate	Standard error	t	Prob(t)
Gamma	1E-005	1.69342128	0	1.0E+030	0.00001
Beta	0.001	0.000271912627	0	1.0E+030	0.00001
s	6000	7301.4638	0	1.0E+030	0.00001
Analysis of Variance					
Source	DF	Sum of Squares	Mean Square	F value	Prob(F)
Regression	2	70975.79	35487.9	112.64	0.00001
Error	32	10081.75	315.0547		
Total	34	81057.54			

Unable to compute confidence intervals because the covariance matrix could not be computed.

The best estimates for the parameters are

$s_c = 7301.4638, \quad \beta_{eff} = 0.000271912627, \quad \gamma_{c,eff} = 1.69342128.$

Using such values, we obtain that $R_0^2 = 1.172395922$.

Second outbreak

Descriptive Statistics for Variables					
Variable	Minimum value	Maximum value	Mean value	Standard dev.	
Mes	1	17	9	5.049752	
TC	20	142	82	40.02031	
Calculated Parameter Values					

Parameter	Initial guess	Final estimate	Standard error	t	Prob(t)
Gamma	1E-005	16.7143009	0	1.0E+030	0.00001
Beta	0.001	0.000383782873	0	1.0E+030	0.00001
s	6000	44325.8656	0	1.0E+030	0.00001
Analysis of Variance					
Source	DF	Sum of Squares	Mean Square	F value	Prob(F)
Regression	2	20964.59	10482.3	31.48	0.00001
Error	14	4661.407	332.9576		
Total	16	25626			

Unable to compute confidence intervals because the covariance matrix could not be computed.
The best estimates for the parameters are

$s_c = 44325.8656, \qquad \beta_{eff} = 0.000383782873, \qquad \gamma_{c,eff} = 16.7143009.$

Using such values, we obtain that $R_0^2 = 1.017781608.$

S.4. Codes for Spain case

S.4.1. Code for estimation using the observed curve of accumulated cases

We use the following code for NLREG:

```
Title "Casos acumulados de Leishmaniasis";
Variables Mes, TC;
Parameters gamma=0.00001, beta=0.0000001, s=1000000;
Double rho, alpha, phi;
rho=gamma/beta;
alpha = ((s/rho-1)^2+2*s/rho^2)^(1/2);
phi =1/2*ln((alpha*rho+s-rho)/(alpha*rho-s+rho));

Function TC =rho^2/s*(s/rho-1-alpha*tanh(-.5*alpha*gamma*Mes+phi));


Plot xvar=Mes, xlabel="Time (Mes)", ylabel="No casos acumulados";
Plot;
rplot;

CONFIDENCE 90;

Data;

7 1
8 3
9 4
10 5
11 5
12 5
13 9
14 11
15 16
16 19
```

```
17 21
18 23
19 33
20 42
21 54
22 70
23 82
24 98
25 129
26 145
27 158
28 171
29 183
30 190
31 199
32 212
33 225
34 234
35 243
36 252
37 256
38 260
39 266
```

Executing such code, we obtain the following results

```
Title "Casos acumulados de Leishmaniasis";
Variables Mes, TC;
Parameters gamma=0.00001, beta=0.0000001, s=1000000;
Double rho, alpha, phi;
rho=gamma/beta;
alpha = ((s/rho-1)^2+2*s/rho^2)^(1/2);
phi =1/2*ln((alpha*rho+s-rho)/(alpha*rho-s+rho));
Function TC =rho^2/s*(s/rho-1-alpha*tanh(-.5*alpha*gamma*Mes+phi));
Plot xvar=Mes, xlabel="Time (Mes)", ylabel="No casos acumulados";
Plot;
rplot;
 CONFIDENCE 90;
Data;
```

```
Beginning computation...
Stopped due to: Relative function convergence.

   ---- Final Results ----

NLREG version 6.5
Copyright (c) 1992-2010 Phillip H. Sherrod.  All rights reserved.
This is a registered copy of NLREG that may not be redistributed.

Casos acumulados de Leishmaniasis
Number of observations = 33
Maximum allowed number of iterations = 500
Convergence tolerance factor = 1.000000E-010
Stopped due to: Relative function convergence.
Number of iterations performed = 140
Final sum of squared deviations = 8.4495041E+002
Final sum of deviations = -1.0135371E+001
Standard error of estimate = 5.30707
Average deviation = 3.71536
Maximum deviation for any observation = 12.4725
Proportion of variance explained (R^2) = 0.9973 (99.73%)
Adjusted coefficient of multiple determination (Ra^2) = 0.9971 (99.71%)
Durbin-Watson test for autocorrelation = 0.518
This Durbin-Watson value indicates autocorrelation or inappropriate function.
Analysis completed 12-May-2017 14:51. Runtime = 0.04 seconds.
```

S.4.2. Code for estimation using the observed incidence curve (Method 1)

In this case we use the following NLREG code:

```
Title "Curva de casos nuevos de Leishmaniasis reportados mensualmente";
Variables Mes, INC;
Parameters gamma=0.0000000001, beta=0.000000001, s=1900;
Double rho, alpha, phi;
rho=gamma/beta;
alpha = ((s/rho-1)^2+2*s/rho^2)^(1/2);
phi =1/2*ln((alpha*rho+s-rho)/(alpha*rho-s+rho));

Function INC =((rho^2)*gamma*(alpha^2)/(2*s))*(cosh(-.5*alpha*gamma*Mes+phi))^(-2);

Plot xvar=Mes xlabel="Time (Mes)", ylabel="Número de casos nuevos por mes";
Plot;
rplot;

 CONFIDENCE 95;

Data;
```

7 1
8 2
9 1
10 1
11 0
12 0
13 4
14 2
15 5
16 3
17 2
18 2
19 10
20 9
21 12
22 16
23 12
24 16
25 31
26 16
27 13
28 13
29 12
30 7
31 9
32 13
33 13
34 9
35 9
36 9
37 4
38 4
39 6

Executing such code, we obtain the following results

```
Title "Curva de casos nuevos de Leishmaniasis reportados mensualmente";
Variables Mes, INC;
Parameters gamma=0.0000000001, beta=0.000000001, s=1900;
Double rho, alpha, phi;
rho=gamma/beta;
alpha = ((s/rho-1)^2+2*s/rho^2)^(1/2);
phi =1/2*ln((alpha*rho+s-rho)/(alpha*rho-s+rho));

Function INC =((rho^2)*gamma*(alpha^2)/(2*s))*(cosh(-.5*alpha*gamma*Mes+phi))^(-2);
```

```
14: Plot xvar=Mes xlabel="Time (Mes)", ylabel="Número de casos nuevos por mes";
15: Plot;
16: rplot;
17:
18:  CONFIDENCE 95;
19:
20: Data;

Beginning computation...

Error executing line 8: phi =1/2*ln((alpha*rho+s-rho)/(alpha*rho-s+rho));
Error: Attempt to take log of zero or negative: -1237.72
Stopped due to: Relative function convergence.

   ---- Final Results ----

NLREG version 6.5
Copyright (c) 1992-2010 Phillip H. Sherrod.  All rights reserved.
This is a registered copy of NLREG that may not be redistributed.

Curva de casos nuevos de Leishmaniasis reportados mensualmente
Number of observations = 33
Maximum allowed number of iterations = 500
Convergence tolerance factor = 1.000000E-010
Stopped due to: Relative function convergence.
Number of iterations performed = 29
Final sum of squared deviations = 4.2924779E+002
Final sum of deviations = 1.2816608E+000
Standard error of estimate = 3.78263
Average deviation = 2.47255
Maximum deviation for any observation = 14.5582
Proportion of variance explained (R^2) = 0.6898 (68.98%)
Adjusted coefficient of multiple determination (Ra^2) = 0.6691 (66.91%)
Durbin-Watson test for autocorrelation = 1.576
Analysis completed 14-May-2017 18:27. Runtime = 0.04 seconds.
```

S.4.3. Code for estimation using the observed curve of accumulated cases (Method 2)

In this case we use the following NLREG code:

```
Title "Curva de casos nuevos de Leishmaniasis reportados mensualmente";
Variables Mes, TC;
Parameters gamma=0.00001, beta=0.00000000000001, s=6000;
Double rho, alpha, phi;
rho=gamma/beta;
alpha = ((s/rho-1)^2+2*s/rho^2)^(1/2);
phi =1/2*ln((alpha*rho+s-rho)/(alpha*rho-s+rho));
```

```
Function TC =rho^2/s*(s/rho-1-alpha*tanh(-.5*alpha*gamma*Mes+phi)) - rho^2/s*(s/rho-1-alpha*tanh(-.5*alpha*gamma*(Mes-1)+phi));

Plot xvar=Mes xlabel="Time (Mes)", ylabel="Número de casos nuevos por mes";
Plot;
rplot;

 CONFIDENCE 95;

Data;

7 1
8 2
9 1
10 1
11 0
12 0
13 4
14 2
15 5
16 3
17 2
18 2
19 10
20 9
21 12
22 16
23 12
24 16
25 31
26 16
27 13
28 13
29 12
30 7
31 9
32 13
33 13
34 9
35 9
36 9
37 4
38 4
39 6
```

Executing such code, we obtain the following results

```
1:
  2: Title "Curva de casos nuevos de Leishmaniasis reportados mensualmente";
```

```
3: Variables Mes, TC;
4: Parameters gamma=0.00001, beta=0.00000000000001, s=6000;
5: Double rho, alpha, phi;
6: rho=gamma/beta;
7: alpha = ((s/rho-1)^2+2*s/rho^2)^(1/2);
8: phi =1/2*ln((alpha*rho+s-rho)/(alpha*rho-s+rho));
9:
10: Function TC =rho^2/s*(s/rho-1-alpha*tanh(-.5*alpha*gamma*Mes+phi)) - rho^2/s*(s/rho-1-alpha*tanh(-.5*alpha*gamma*(Mes-1)+phi));
11:
12:
13:
14: Plot xvar=Mes xlabel="Time (Mes)", ylabel="Número de casos nuevos por mes";
15: Plot;
16: rplot;
17:
18:  CONFIDENCE 95;
19:
20: Data;
```

```
Beginning computation...
Stopped due to: Relative function convergence.
  ---- Final Results ----

NLREG version 6.5
Copyright (c) 1992-2010 Phillip H. Sherrod.  All rights reserved.
This is a registered copy of NLREG that may not be redistributed.

Curva de casos nuevos de Leishmaniasis reportados mensualmente
Number of observations = 33
Maximum allowed number of iterations = 500
Convergence tolerance factor = 1.000000E-010
Stopped due to: Relative function convergence.
Number of iterations performed = 41
Final sum of squared deviations = 4.2927268E+002
Final sum of deviations = 1.2837181E+000
Standard error of estimate = 3.78274
Average deviation = 2.4721
Maximum deviation for any observation = 14.5614
Proportion of variance explained (R^2) = 0.6898 (68.98%)
Adjusted coefficient of multiple determination (Ra^2) = 0.6691 (66.91%)
Durbin-Watson test for autocorrelation = 1.576
Analysis completed 14-May-2017 18:49. Runtime = 0.04 seconds.
```

S.5. Codes for Colombia case

S.5.1. Code for estimation using the observed curve of accumulated cases

<u>First outbreak</u>

```
Title "Casos acumulados de Leishmaniasis";
Variables Semana, TC;
Parameters gamma=0.00001, beta=0.0000001, s=1000000;
Double rho, alpha, phi;
rho=gamma/beta;
alpha = ((s/rho-1)^2+2*s/rho^2)^(1/2);
phi =1/2*ln((alpha*rho+s-rho)/(alpha*rho-s+rho));

Function TC =rho^2/s*(s/rho-1-alpha*tanh(-.5*alpha*gamma*Semana+phi));



Plot xvar=Semana, xlabel="Time (Semana)", ylabel="No casos acumulados";
Plot;
rplot;


 CONFIDENCE 90;

Data;

1  1
2  2
3  5
4  10
5  14
6  15
7  21
8  29
9  29
10   50
11 74
12  85
13  145
14  210
15  309
16  413
17  538
18  659
19  794
20  939
21  1032
22 1170
23 1303
24  1381
25  1424
26  1534
27  1591
```

```
28  1649
29  1671
30  1703
31  1729
32  1743
33  1770
34  1788
35  1825
```

Executing such code, we obtain the following results

```
1:
  2: Title "Casos acumulados de Leishmaniasis";
  3: Variables Semana, TC;
  4: Parameters gamma=0.00001, beta=0.0000001, s=1000000;
  5: Double rho, alpha, phi;
  6: rho=gamma/beta;
  7: alpha = ((s/rho-1)^2+2*s/rho^2)^(1/2);
  8: phi =1/2*ln((alpha*rho+s-rho)/(alpha*rho-s+rho));
  9:
 10: Function TC =rho^2/s*(s/rho-1-alpha*tanh(-.5*alpha*gamma*Semana+phi));
 11:
 12:
 13:
 14: Plot xvar=Semana, xlabel="Time (Semana)", ylabel="No casos acumulados";
 15: Plot;
 16: rplot;
 17:
 18:
 19:
 20:  CONFIDENCE 90;
 21:
 22: Data;
```

```
Beginning computation...
Stopped due to: Relative function convergence.

   ---- Final Results ----

NLREG version 6.5
Copyright (c) 1992-2010 Phillip H. Sherrod.  All rights reserved.
This is a registered copy of NLREG that may not be redistributed.

Casos acumulados de Leishmaniasis
Number of observations = 35
Maximum allowed number of iterations = 500
Convergence tolerance factor = 1.000000E-010
Stopped due to: Relative function convergence.
Number of iterations performed = 102
```

Final sum of squared deviations = 1.9381341E+004
Final sum of deviations = -1.4892068E+002
Standard error of estimate = 24.6103
Average deviation = 17.5432
Maximum deviation for any observation = 57.1837
Proportion of variance explained (R^2) = 0.9989 (99.89%)
Adjusted coefficient of multiple determination (Ra^2) = 0.9988 (99.88%)
Durbin-Watson test for autocorrelation = 0.542
This Durbin-Watson value indicates autocorrelation or inappropriate function.

Warning: Covariance matrix could not be computed because
the finite-difference Hessian was indefinite.

Analysis completed 18-May-2017 16:20. Runtime = 0.05 seconds.

Second outbreak

```
Title "Casos acumulados de Leishmaniasis";
Variables Semana, TC;
Parameters gamma=0.00001, beta=0.0000001, s=1000;
Double rho, alpha, phi;
rho=gamma/beta;
alpha = ((s/rho-1)^2+2*s/rho^2)^(1/2);
phi =1/2*ln((alpha*rho+s-rho)/(alpha*rho-s+rho));

Function TC =rho^2/s*(s/rho-1-alpha*tanh(-.5*alpha*gamma*Semana+phi));


Plot xvar=Semana, xlabel="Time (Semana)", ylabel="No casos acumulados";
Plot;
rplot;

 CONFIDENCE 90;

Data;


1  20
2  56
3  93
4  135
5  199
6  299
7  349
8  443
9  561
10   703
11  827
12  938
13  1080
```

```
14  1181
15  1272
16  1363
17  1394
18  1396
19  1398
```

Executing such code, we obtain the following results

```
Title "Casos acumulados de Leishmaniasis";
Variables Semana, TC;
Parameters gamma=0.00001, beta=0.0000001, s=1000;
Double rho, alpha, phi;
rho=gamma/beta;
alpha = ((s/rho-1)^2+2*s/rho^2)^(1/2);
phi =1/2*ln((alpha*rho+s-rho)/(alpha*rho-s+rho));

Function TC =rho^2/s*(s/rho-1-alpha*tanh(-.5*alpha*gamma*Semana+phi));

Plot xvar=Semana, xlabel="Time (Semana)", ylabel="No casos acumulados";
Plot;
rplot;

 CONFIDENCE 90;

Data;
```

Beginning computation...
Stopped due to: Both parameter and relative function convergence.

---- Final Results ----

NLREG version 6.5
Copyright (c) 1992-2010 Phillip H. Sherrod. All rights reserved.
This is a registered copy of NLREG that may not be redistributed.

Casos acumulados de Leishmaniasis
Number of observations = 19
Maximum allowed number of iterations = 500
Convergence tolerance factor = 1.000000E-010
Stopped due to: Both parameter and relative function convergence.
Number of iterations performed = 109
Final sum of squared deviations = 8.0222411E+003
Final sum of deviations = 4.3831294E+001

Standard error of estimate = 22.3917
Average deviation = 16.407
Maximum deviation for any observation = 43.7928
Proportion of variance explained (R^2) = 0.9983 (99.83%)
Adjusted coefficient of multiple determination (Ra^2) = 0.9981 (99.81%)
Durbin-Watson test for autocorrelation = 0.898

Warning: Covariance matrix could not be computed because
the finite-difference Hessian was indefinite.

Analysis completed 19-May-2017 06:05. Runtime = 0.03 seconds.

S.5.2. Code for estimation using the observed incidence curve (Method 1)

<u>First outbreak</u>

```
Title "Curva de casos nuevos de Leishmaniasis reportados semanalmente";
Variables Semana, INC;
Parameters gamma=0.0000000001, beta=0.000000001, s=1900;
Double rho, alpha, phi;
rho=gamma/beta;
alpha = ((s/rho-1)^2+2*s/rho^2)^(1/2);
phi =1/2*ln((alpha*rho+s-rho)/(alpha*rho-s+rho));

Function INC =((rho^2)*gamma*(alpha^2)/(2*s))*(cosh(-.5*alpha*gamma*Semana+phi))^(-2);


Plot xvar=Semana xlabel="Time (Semana)", ylabel="Número de casos nuevos por semana";
Plot;
rplot;

 CONFIDENCE 95;

Data;

1  1
2  1
3  3
4  5
5  4
6  1
7  6
8  8
9  0
10  21
11  24
12  9
13  60
14  65
```

```
15  99
16  104
17  125
18  121
19  135
20  145
21  93
22  138
23  133
24  78
25  43
26  110
27  57
28  58
29  22
30  32
31  26
32  14
33  27
34  18
35  38
```

Executing such code, we obtain the following results

```
Title "Curva de casos nuevos de Leishmaniasis reportados semanalmente";
Variables Semana, INC;
Parameters gamma=0.0000000001, beta=0.000000001, s=1900;
Double rho, alpha, phi;
rho=gamma/beta;
alpha = ((s/rho-1)^2+2*s/rho^2)^(1/2);
phi =1/2*ln((alpha*rho+s-rho)/(alpha*rho-s+rho));

Function INC =((rho^2)*gamma*(alpha^2)/(2*s))*(cosh(-.5*alpha*gamma*Semana+phi))^(-2);

Plot xvar=Semana xlabel="Time (Semana)", ylabel="Número de casos nuevos por semana";
Plot;
rplot;

CONFIDENCE 95;

Data;
```

Beginning computation...
Stopped due to: Relative function convergence.

---- Final Results ----

```
NLREG version 6.5
Copyright (c) 1992-2010 Phillip H. Sherrod.  All rights reserved.
This is a registered copy of NLREG that may not be redistributed.

Curva de casos nuevos de Leishmaniasis reportados semanalmente
Number of observations = 35
Maximum allowed number of iterations = 500
Convergence tolerance factor = 1.000000E-010
Stopped due to: Relative function convergence.
Number of iterations performed = 21
Final sum of squared deviations = 1.0083147E+004
Final sum of deviations = 6.2758196E+000
Standard error of estimate = 17.751
Average deviation = 12.1321
Maximum deviation for any observation = 41.0307
Proportion of variance explained (R^2) = 0.8756 (87.56%)
Adjusted coefficient of multiple determination (Ra^2) = 0.8678 (86.78%)
Durbin-Watson test for autocorrelation = 2.100

Warning: Covariance matrix could not be computed because
the finite-difference Hessian was indefinite.

Analysis completed 19-May-2017 10:03.  Runtime = 0.04 seconds.
```

Second outbreak

```
Title "Curva de casos nuevos de Leishmaniasis reportados semanalmente";
Variables Semana, INC;
Parameters gamma=0.0000000001, beta=0.000000001, s=1900;
Double rho, alpha, phi;
rho=gamma/beta;
alpha = ((s/rho-1)^2+2*s/rho^2)^(1/2);
phi =1/2*ln((alpha*rho+s-rho)/(alpha*rho-s+rho));

Function INC =((rho^2)*gamma*(alpha^2)/(2*s))*(cosh(-.5*alpha*gamma*Semana+phi))^(-2);

Plot xvar=Semana xlabel="Time (Semana)", ylabel="Número de casos nuevos por semana";
Plot;
rplot;

 CONFIDENCE 95;

Data;


1  20
2  36
3  37
```

```
4  42
5  64
6  100
7  50
8  94
9  118
10  142
11  124
12  111
13  142
14  101
15  91
16  91
17  31
```

Executing such code, we obtain the following results

```
Title "Curva de casos nuevos de Leishmaniasis reportados semanalmente";
Variables Semana, INC;
Parameters gamma=0.0000000001, beta=0.000000001, s=1900;
Double rho, alpha, phi;
rho=gamma/beta;
alpha = ((s/rho-1)^2+2*s/rho^2)^(1/2);
phi =1/2*ln((alpha*rho+s-rho)/(alpha*rho-s+rho));

Function INC =((rho^2)*gamma*(alpha^2)/(2*s))*(cosh(-.5*alpha*gamma*Semana+phi))^(-2);



Plot xvar=Semana xlabel="Time (Semana)", ylabel="Número de casos nuevos por semana";
Plot;
rplot;

 CONFIDENCE 95;

Data;
```

Beginning computation...
Stopped due to: Relative function convergence.

---- Final Results ----

NLREG version 6.5
Copyright (c) 1992-2010 Phillip H. Sherrod. All rights reserved.
This is a registered copy of NLREG that may not be redistributed.

Curva de casos nuevos de Leishmaniasis reportados semanalmente
Number of observations = 17

```
Maximum allowed number of iterations = 500
Convergence tolerance factor = 1.000000E-010
Stopped due to: Relative function convergence.
Number of iterations performed = 60
Final sum of squared deviations = 4.6603509E+003
Final sum of deviations = 4.6897275E+000
Standard error of estimate = 18.2451
Average deviation = 12.3967
Maximum deviation for any observation = 37.4176
Proportion of variance explained (R^2) = 0.8181 (81.81%)
Adjusted coefficient of multiple determination (Ra^2) = 0.7922 (79.22%)
Durbin-Watson test for autocorrelation = 2.452

Warning: Covariance matrix could not be computed because
the finite-difference Hessian was indefinite.

Analysis completed 19-May-2017 10:13. Runtime = 0.04 seconds.
```

S.5.3. Code for estimation using the observed curve of accumulated cases (Method 2)

<u>First outbreak</u>

```
Title "Curva de casos nuevos de Leishmaniasis reportados semanalmente";
Variables Semana, TC;
Parameters gamma=0.00001, beta=0.001, s=6000;
Double rho, alpha, phi;
rho=gamma/beta;
alpha = ((s/rho-1)^2+2*s/rho^2)^(1/2);
phi =1/2*ln((alpha*rho+s-rho)/(alpha*rho-s+rho));

Function TC =rho^2/s*(s/rho-1-alpha*tanh(-.5*alpha*gamma*Semana+phi)) - rho^2/s*(s/rho-1-alpha*tanh(-.5*alpha*gamma*(Semana-1)+phi));


Plot xvar=Semana xlabel="Time (Mes)", ylabel="Número de casos nuevos por semana";
Plot;
rplot;

 CONFIDENCE 95;

Data;

1  1
2  1
3  3
4  5
5  4
```

```
6  1
7  6
8  8
9  0
10  21
11  24
12  9
13  60
14  65
15  99
16  104
17  125
18  121
19  135
20  145
21  93
22  138
23  133
24  78
25  43
26  110
27  57
28  58
29  22
30  32
31  26
32  14
33  27
34  18
35  38
```

Executing such code, we obtain the following results

```
Title "Curva de casos nuevos de Leishmaniasis reportados semanalmente";
Variables Semana, TC;
Parameters gamma=0.00001, beta=0.001, s=6000;
Double rho, alpha, phi;
rho=gamma/beta;
alpha = ((s/rho-1)^2+2*s/rho^2)^(1/2);
phi =1/2*ln((alpha*rho+s-rho)/(alpha*rho-s+rho));
Function TC =rho^2/s*(s/rho-1-alpha*tanh(-.5*alpha*gamma*Semana+phi)) - rho^2/s*(s/rho-1-alpha*tanh(-.5*alpha*gamma*(Semana-1)+phi));
Plot xvar=Semana xlabel="Time (Mes)", ylabel="Número de casos nuevos por semana";
Plot;
```

```
16: rplot;
17:
18:  CONFIDENCE 95;
19:
20: Data;
```

Beginning computation...
Stopped due to: Relative function convergence.

---- Final Results ----

NLREG version 6.5
Copyright (c) 1992-2010 Phillip H. Sherrod. All rights reserved.
This is a registered copy of NLREG that may not be redistributed.

Curva de casos nuevos de Leishmaniasis reportados semanalmente
Number of observations = 35
Maximum allowed number of iterations = 500
Convergence tolerance factor = 1.000000E-010
Stopped due to: Relative function convergence.
Number of iterations performed = 49
Final sum of squared deviations = 1.0081751E+004
Final sum of deviations = 6.5011182E+000
Standard error of estimate = 17.7498
Average deviation = 12.1288
Maximum deviation for any observation = 41.0568
Proportion of variance explained (R^2) = 0.8756 (87.56%)
Adjusted coefficient of multiple determination (Ra^2) = 0.8678 (86.78%)
Durbin-Watson test for autocorrelation = 2.100

Warning: Covariance matrix could not be computed because
the finite-difference Hessian was indefinite.

Analysis completed 19-May-2017 14:44. Runtime = 0.05 seconds.

Second outbreak

```
Title "Curva de casos nuevos de Leishmaniasis reportados semanalmente";
Variables Semana, TC;
Parameters gamma=0.00001, beta=0.001, s=6000;
Double rho, alpha, phi;
rho=gamma/beta;
alpha = ((s/rho-1)^2+2*s/rho^2)^(1/2);
phi =1/2*ln((alpha*rho+s-rho)/(alpha*rho-s+rho));

Function TC =rho^2/s*(s/rho-1-alpha*tanh(-.5*alpha*gamma*Semana+phi)) - rho^2/s*(s/rho-1-alpha*tanh(-.5*alpha*gamma*(Semana-1)+phi));


Plot xvar=Semana xlabel="Time (Semana)", ylabel="Número de casos nuevos por semana";
```

```
Plot;
rplot;

 CONFIDENCE 95;

Data;

1  20
2  36
3  37
4  42
5  64
6  100
7  50
8  94
9  118
10  142
11  124
12  111
13  142
14  101
15  91
16  91
17  31
```

Executing such code, we obtain the following results

```
1:
  2: Title "Curva de casos nuevos de Leishmaniasis reportados semanalmente";
  3: Variables Semana, TC;
  4: Parameters gamma=0.00001, beta=0.001, s=6000;
  5: Double rho, alpha, phi;
  6: rho=gamma/beta;
  7: alpha = ((s/rho-1)^2+2*s/rho^2)^(1/2);
  8: phi =1/2*ln((alpha*rho+s-rho)/(alpha*rho-s+rho));
  9:
 10: Function TC =rho^2/s*(s/rho-1-alpha*tanh(-.5*alpha*gamma*Semana+phi))  -  rho^2/s*(s/rho-1-
alpha*tanh(-.5*alpha*gamma*(Semana-1)+phi));
 11:
 12:
 13:
 14: Plot xvar=Semana xlabel="Time (Semana)", ylabel="Número de casos nuevos por semana";
 15: Plot;
 16: rplot;
 17:
 18:  CONFIDENCE 95;
 19:
 20: Data;
```

Beginning computation...
Stopped due to: Relative function convergence.

---- Final Results ----

NLREG version 6.5
Copyright (c) 1992-2010 Phillip H. Sherrod. All rights reserved.
This is a registered copy of NLREG that may not be redistributed.

Curva de casos nuevos de Leishmaniasis reportados semanalmente
Number of observations = 17
Maximum allowed number of iterations = 500
Convergence tolerance factor = 1.000000E-010
Stopped due to: Relative function convergence.
Number of iterations performed = 75
Final sum of squared deviations = 4.6614070E+003
Final sum of deviations = 4.7134677E+000
Standard error of estimate = 18.2471
Average deviation = 12.3987
Maximum deviation for any observation = 37.4434
Proportion of variance explained (R^2) = 0.8181 (81.81%)
Adjusted coefficient of multiple determination (Ra^2) = 0.7921 (79.21%)
Durbin-Watson test for autocorrelation = 2.452

Warning: Covariance matrix could not be computed because
the finite-difference Hessian was indefinite.

Analysis completed 19-May-2017 14:56. Runtime = 0.04 seconds.
